# Evaluation and selection of internal reference genes from two- and six-row U.S. malting barley varieties throughout micromalting for use in RT-qPCR

**DOI:** 10.1371/journal.pone.0196966

**Published:** 2018-05-08

**Authors:** Jason G. Walling, Leslie A. Zalapa, Marcus A. Vinje

**Affiliations:** Cereal Crops Research Unit, Agricultural Research Service, United States Department of Agriculture, Madison, Wisconsin, United States of America; Northwestern University Feinberg School of Medicine, UNITED STATES

## Abstract

Reverse transcription quantitative polymerase chain reaction (RT-qPCR) is a popular method for measuring transcript abundance. The most commonly used method of interpretation is relative quantification and thus necessitates the use of normalization controls (i.e. reference genes) to standardize transcript abundance. The most popular gene targets for RT-qPCR are housekeeping genes because they are thought to maintain a static transcript level among a variety of samples. However, more recent studies have shown, several housekeeping genes are not reliably stable. This is the first study to examine the potential of several reference genes for use in RT-qPCR normalization during barley malting. The process of malting barley mechanizes the imbibition and subsequent germination of barley seeds under controlled conditions. Malt quality is controlled by many pleiotropic genes that are determined by examining the result of physiological changes the barley seed undergoes during the malting process. We compared the stability of 13 reference genes across both two-and six-row malting barleys (Conrad and Legacy, respectfully) throughout the entirety of the malting process. Initially, primer target specificity, amplification efficiency and average Ct values were determined for each of the selected primer pairs. Three statistical programs (geNorm, NormFinder, and BestKeeper) were used to rank the stability of each reference gene. Rankings were similar between the two- and six-row with the exception of BestKeeper’s ranking of glyceraldehyde-3-phosphate dehydrogenase (*GAPDH*). A consensus ranking among programs was determined using RefFinder. Our results show that Actin (A*CT*) and Heat Shock Protein 70 (*HSP70*) were the most stable throughout micromalting, while *GAPDH* and Cyclophilin (*CYP*) were the least stable. Two reference genes are necessary for stable transcript normalization according to geNorm and the best two reference genes (*ACT* and *HSP70*) provided a sufficient level of stability.

## Introduction

Reverse-transcription quantitative polymerase chain reaction (RT-qPCR) is commonly used to monitor gene expression by measuring mRNA levels. RT-qPCR is an extremely powerful method to accurately quantify gene transcripts but the power behind this method is in the experimental design and properly chosen stable reference genes. Plant housekeeping genes, such as actin, tubulin, heat shock proteins, and rRNA, have been used as internal reference genes representing stable gene expression because they were historically used for non-quantitative methods such as Northern Blots [1,2]. The stability of internal reference genes must be verified for each experiment because genotypes, qPCR reaction conditions and consumables, primer sequences, and numerous other factors can have an effect on the stability of the reference gene chosen and thus, poorly chosen reference genes can yield erroneous results [3,4]. It is entirely too commonplace for RT-qPCR data to be reported without proper validation of the internal reference genes used, which has been addressed by numerous reports outlining the need for standard reporting procedures such as the MIQE guidelines [5,6]. The quality of the RT-qPCR data is influenced by numerous factors including, but not limited to, sample preparation, RNA integrity, reverse transcriptase (e.g. SuperScript III vs iScript), primer set efficiency, and qPCR chemistry [3,5].

The US grows barley primarily for beer (65%) and feed (27%) but also uses barley for food (3%), seed (3%), and whiskey (2%) production [7]. The majority of barley used in making alcohol, food, or other industry applications is first malted. Malting is the controlled germination of barley and consists of steeping (imbibition of water), germination, and kilning (controlled drying of malt). In beer production, malted barley is milled and combined with warm to hot water, collectively called a mash, to create a sweet wort, which is fermented by the addition of yeast to produce beer. The production of sweet wort depends on the ability of the hydrolytic enzymes in the malt to break down the starches to produce smaller, fermentable sugars.

Barley row-type is determined by lateral spikelet fertility and this difference has measureable effects on the agronomic qualities of the grain. Two-rowed barley has infertile lateral spikelets whereas six-rowed barley has fertile lateral spikelets [8]. In general, two-row malting varieties produce larger seeds, lower wort β-glucans, lower protein by all measures, lower diastatic power (DP), higher α-amylase activity, and higher malt extract, and is generally considered easier to use in brewing [9,10]. Historically, six-row barley varieties were the predominant row-type used by the malting and brewing industries in the U.S. (e.g. Six-row was 75% in 1996) but since 2010 two-row barley is being used for malt at higher rates than six-row barley (Two row was 65.6% in 2015) [11,12]. Six-rowed malt is commonly used by companies brewing adjunct lagers that require higher levels of malt enzymes to breakdown the added starch (i.e. adjunct).

Identification of stable internal reference genes in two- and six-row U.S. malting barley varieties is beneficial to barley researchers who study the biology of malting and germination. Considering the simplicity and power of RT-qPCR, this technique could be employed by malting and brewing industry laboratories and potentially added to their quality control programs. The most labor-intensive portion of a gene expression experiment is commonly identifying stable reference genes throughout your experimental conditions. The data presented here allow for malting brewing researchers and perhaps in the future technical laboratories to use this powerful assay to identify changes in genes of interest during malting. Furthermore, the establishment of stable internal reference genes is of great importance in modern gene expression studies because RT-qPCR is normally used to validate differentially expressed genes identified using RNAseq or microarrays. In order to determine quality stable internal reference genes for use in RT-qPCR and circumvent the confluences of genotype by environment ten biological replications representing two malting cultivars (two-row U.S. malting barley variety Conrad and six-row U.S. malting barley variety Legacy) were grown in four different locations over four crop years and subsequently micromalted. Thirteen internal reference genes were tested throughout micromalting and all thirteen primer sets studied were determined to be acceptable using three popular software packages (GeNorm, BestKeeper, and NormFinder). Additionally, the top two stable reference genes were identified along with a consensus among the three software packages was determined using RefFinder.

## Materials and methods

### Plant material and malting

Seeds from the two-row malting cultivar Conrad and the six-row malting cultivar Legacy were grown and collected from four unique environments grown over four crop years (2012–2015). The four locations were Aberdeen, ID (42° 56’ N, 112° 50’ W), Morris, MN (45° 35’ N, 95° 55’ W), Crookston, MN (47° 46’ N, 96° 36’ W), and Fargo, ND (46° 52’ N, 96°, 47’ W) (S1 Table).

One hundred and ten grams on a dry basis of each barley sample were micromalted at the Cereal Crops Research Unit’s Malt Quality lab (Madison, WI) in adherence to the methods recommended by the American Society of Brewing Chemists [13]. In short, the malting procedure consisted of the imbibition of barley grains (i.e. steeping) in a tank that periodically submerged the barley over a 36-hour period, which homogenized the barley at a specific moisture content. After the steep, the imbibed barley seeds were transferred to germinators and incubated under controlled temperature (16 °C), humidity (95%), and airflow conditions for five days.

The sampling time points will be defined heretofore as Days of Germination (DoG) 0–5; i.e. DoG 0 (Out of Steep), DoG #1, DoG#2, DoG#3, DoG#4 and DoG#5. Fifty grams of each of the barleys were sampled every 24 hours throughout the malting process, immediately flash frozen in liquid N_2_, and stored at -80 °C (S1 Fig).

### RNA isolation, quality check and cDNA synthesis

Total RNA was isolated from seed sampled from malting stages according to Vinje et al. [14]. Briefly, a few seeds were ground in liquid N_2_ and approximately 100 mg of tissue was suspended in Plant RNA Purification Reagent (Invitrogen, Carlsbad, CA). Total RNA isolation was carried out according to the manufacturer’s protocol. The resulting total RNA was further purified using Qiagen RNeasy columns with an on-column DNase I treatment (Valencia, CA).

RNA concentration and integrity were determined using a Nanodrop Spectrophotometer (ThermoScientific Wilmington, DE) and BioAnalyzer (Agilent, Santa Clara, CA), respectively. All extractions yielded over three micrograms of total RNA, had 260/280 ratios of greater than or equal to 2.0 and RNA integrity numbers of greater than or equal to 7.5.

The iScript Advanced cDNA Synthesis kit (BioRad, Hercules, CA) was used to synthesize cDNA from one microgram of total RNA from each of the samples according to the manufacturer’s protocol. Two technical replications of each synthesis were performed on each sample.

Primers were selected as a result of surveying reference primers commonly utilized in RT-qPCR (i.e. housekeeping genes) and a few contemporary reference targets used in other cereal crop experiments. Primer3 Plus (http://primer3plus.com/cgi-bin/dev/primer3plus.cgi) was used to check the primer Tm and secondary structure (Table 1).

**Table 1:** Barley candidate reference genes and primer sequences.

Gene (Abbreviation)	GenBank Accession Number	Forward Primer	Reverse Primer	Amplicon Size (bp)^a^	PCR Efficiency (%)	R2
Actin (ACT)[14]	AY14545.1	GGCATGGAGTCTTCTGGAATCC	CCACCACTGAGCACTATGTTTC	115	102.63	1.00
ADP-ribosylation factor 1-like protein (ADP)[15]	AK365041.1	GCTCTCCAACAACATTGCCAAC	GAGACATCCAGCATCATTCATTCC	77	105.97	0.99
ATP Binding Protein (ABC)[16]	AK365041.1	ATCTGAGGTCTCGGTTCGGA	TCCTTCAGCTGACAACGGTC	116	99.28	1.00
Cyclophilin (CYP)[14]	AK248688.1	CCTGTCGTGTCGTCGGTCTAAA	ACGCAGATCCAGGAGCCTAAAG	122	98.44	1.00
Glyceraldehyde 3-Phosphate Dehydrogenase (GAPDH)[17]	X60343.1	GCCAGTTACTGTCTTTGGCGTC	GGCCTTGTCCTGTCAGTGAAG	108	93.40	1.00
Glycine Rich RNA Binding Protein (GRBP)[17]	Z48624.1	CGCCCAGTTATCCATCCATCTA	AAAAACACCACAGGACCGGAC	112	99.47	1.00
Heat Shock Protein 70 (HSP70)[17]	AK354795.1	GCTCAACATGGACCTCTTCAGG	CCGACAAGGACAACATCATGG	101	96.2	1.00
Heat Shock Protein 90 (HSP90)[17]	AY325266.1	CAAGAAGCTTGTCTCTGCCACC	ACAGCCCCTCGAACTTCTCCTT	100	94.50	1.00
Small Nucleolar RNA 14 (SnoR14)[15]	AK373867.1	GATGTTTATGTATGATAGTCTGTC	GTCGGGATGTATGCGTGTC	67	98.73	1.00
Small Nucleolar RNA 23 (SnoR23)[15]	AK373724.1	TCGGCAGTGGTGTCATC	CTCAGTGGAAAGAGAAGTCG	64	104.63	1.00
Translationally Controlled Tumor Protein (TCTP)[16]	AF230786.1	TTCCGTCTTCAGGAGCAACC	ATGCTCTCGCCCACAAAGAA	177	100.46	1.00
Tubulin Alpha-2 Chain (α-TUB)[15]	U40042.1	GTCCACCCACTCCCTCCTTG	CGGCGGCAGATGTCATAGATG	78	100.77	1.00
Ubiquitin (UBQ)[18]	AK249354.1	TCAAGGTGAAGACACTTACTGG	CATAGATGAGCCTCTGTTGAAC	128	96.71	1.00

^a^Amplicons located in the coding region.

qPCR reactions were performed using the QuantStudio 6 Flex Real-Time PCR System (Applied Biosystems, Life Technologies, USA and SYBR Premix Ex Taq (Takara, Madison, WI) in 96 well Micro-Amp optical reaction plates sealed with optical adhesive film (Applied Biosystems, Life Technologies, USA). Each 20 μl reaction contained 10 μl of SYBR Premix Ex Taq (Tli RNase H Plus) (2X), 0.08 μl of ROX Reference Dye, 0.4 μl of each primer (10 μM), 2 μl of diluted cDNA (1:20) and nuclease-free water. Thermal cycling conditions were 95°C for 30s, 40 cycles of 5s at 95°C and 34s at 60°C. The threshold cycle (Ct) was automatically calculated by the instrument software. All qPCR reactions were carried out using five biological replicates for each time point and two technical replicates from each biological replicate.

The stability of candidate reference genes was analyzed using three software packages: geNorm (version 3.1) (http:biogazelle.com/genormplus/website), NormFinder and BestKeeper. NormFinder and BestKeeper are standalone program modules included in RefFinder (http://leonxie.esy.es/RefFinder), a software package that includes a suite of programs to analyze qPCR data. RefFinder also has the capability of developing an overall consensus ranking of stability by examining the output from each of the three programs (geNorm, NormFinder, BestKeeper), as well an additional stability ranking method (comparative ΔCt) to rank all genes.

## Results and discussion

### Reference gene expression analysis

Each of the 13 reference genes were tested for target specificity as determined by melt curve analysis and amplicon size using gel electrophoresis (S2 Fig). Ten out of thirteen genes produced a single distinct peak in their respective melt curve indicating strong primer specificity allowing for a single amplicon. Three genes (*GRBP*, *HSP90* and SnoR14) displayed various and often inconsistent levels of nonspecific amplification. The secondary amplicons were not observed using gel electrophoresis 0f *GRBP*, *HSP90* and SnoR14 nor do they affect the Ct values (S1 Fig). Ct values from samples with a spurious melt curve peak did not deviate from Ct values of identical samples without the secondary amplicon (data not shown). While the secondary products from these three primers pairs detected from the melt curve are concerning and therefore should be tested for target specificity when deciding whether to use them, we continued to employ them in this study to test whether the secondary amplicons affected the primer sets ability as a reference gene.

Amplification efficiency was assessed for all 13 primer pairs. Each primer pair was used to amplify, in triplicate, cDNA from 2 DoG that was serial diluted five times in 1o-fold iterations. Percent amplification efficiencies for all primer pairs ranged between 93–105 and regression coefficients (R^2^) close to 1.oo (Table 1), demonstrating that primers evaluated here all fall within the efficiency range required for accurate determination of amplification rate [19].

**Fig 1:**
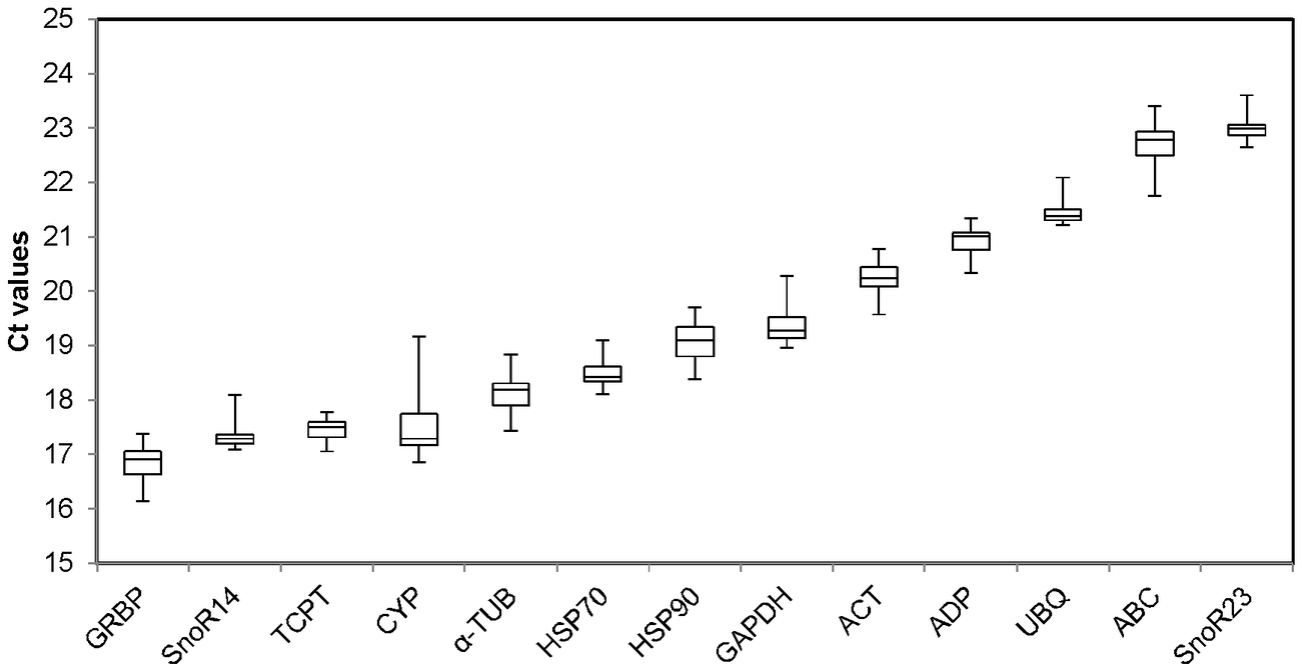
Ct distribution of 13 candidate reference genes. Values are given as the cycle threshold (Ct). Expression levels of the different genes tested are shown as the 25^th^ and 75^th^ quartiles (upper and lower hinges), median (central horizontal lines) and whiskers. Whiskers represent maximum and minimum Ct values. Genes are shown from the most (lower Ct, *left*) to the least abundantly expressed (higher Ct, *right*).

Ct distributions across all two- and six- row micromalted barley samples were plotted for each of the 13 genes (Fig 1). All 13 putative reference genes have average Ct thresholds between 16 and 24 cycles. The genes with the lowest average Ct threshold represent a higher fraction of total mRNA isolated for each respective time point. Here, SnoR14, *TCPT*, *GRBP* and *CYP* all have average Ct thresholds lower than 18 Ct indicating that of the 13 genes assessed, these four have the highest transcript representation in the total mRNA. The genes with the highest average Ct value were SnoR23 and *ABC* which were between 22 and 24 Ct suggesting that these two genes are found in lower abundance during barley malting. The coefficient of variation (CV) of Ct distributions from 12 out of 13 thirteen putative reference genes were less than 3.0 with the exception of *CYP* (Avg 17.57%, CV 3.84). Reference targets for qPCR analysis should have average Ct values within a range shared by most experimental target genes [20] and therefore all reference targets here exhibit an acceptable level of stability across samples.

The Ct values range for each reference target demonstrated variability of target abundance among samples and exemplified the necessity to evaluate reference gene panels for each experimental sample type. Ferdous et al. [15] found that the small nucleolar RNA’s (SNOR14 and SNO23) generated the highest Ct values of RT-qPCR reference genes in barley under stress conditions, however in our micromalted samples SnoR14 was highly expressed while SnoR23 was one of the lowest. *GAPDH* is one of the highly expressed genes among putative reference genes used in these panels [21,22], and the data presented here reflect that phenomenon with *GAPDH* having an average Ct of 18. Given the wide range of transcript representation in cDNA synthesized from total RNA, reference genes that tend to exhibit lower average Ct values generally provide a more robust amplification across most sample types; an attractive characteristic that may shed light on the recurrent popularity of *GAPDH*’s use as a reference in RT-qPCR [23].

### Evaluation of reference gene stability in two- and six- row micromalted barley using GeNorm, BestKeeper and NormFinder

While the distribution of a putative reference gene’s Ct values can be informative in regards to overall gene expression and consistency among experimental samples, we used the three most common computer programs for a precise evaluation of candidate reference gene stability: geNorm, NormFinder, and BestKeeper. The output from each of these programs was used to determine stability rankings of all putative reference genes in both two- and six-row malting barleys and also when considering both row types collectively.

#### GeNorm

GeNorm generates an “M” score by performing pairwise comparisons in stepwise iterations until the most stable reference genes are identified [24]. The M value provides a metric by which to rank the most stable to least, with most stable reference having the smallest “M” value. GeNorm suggests that for a gene to be considered stable across treatment samples, the M value should be lower than 1.5 [24].

**Fig 2:**
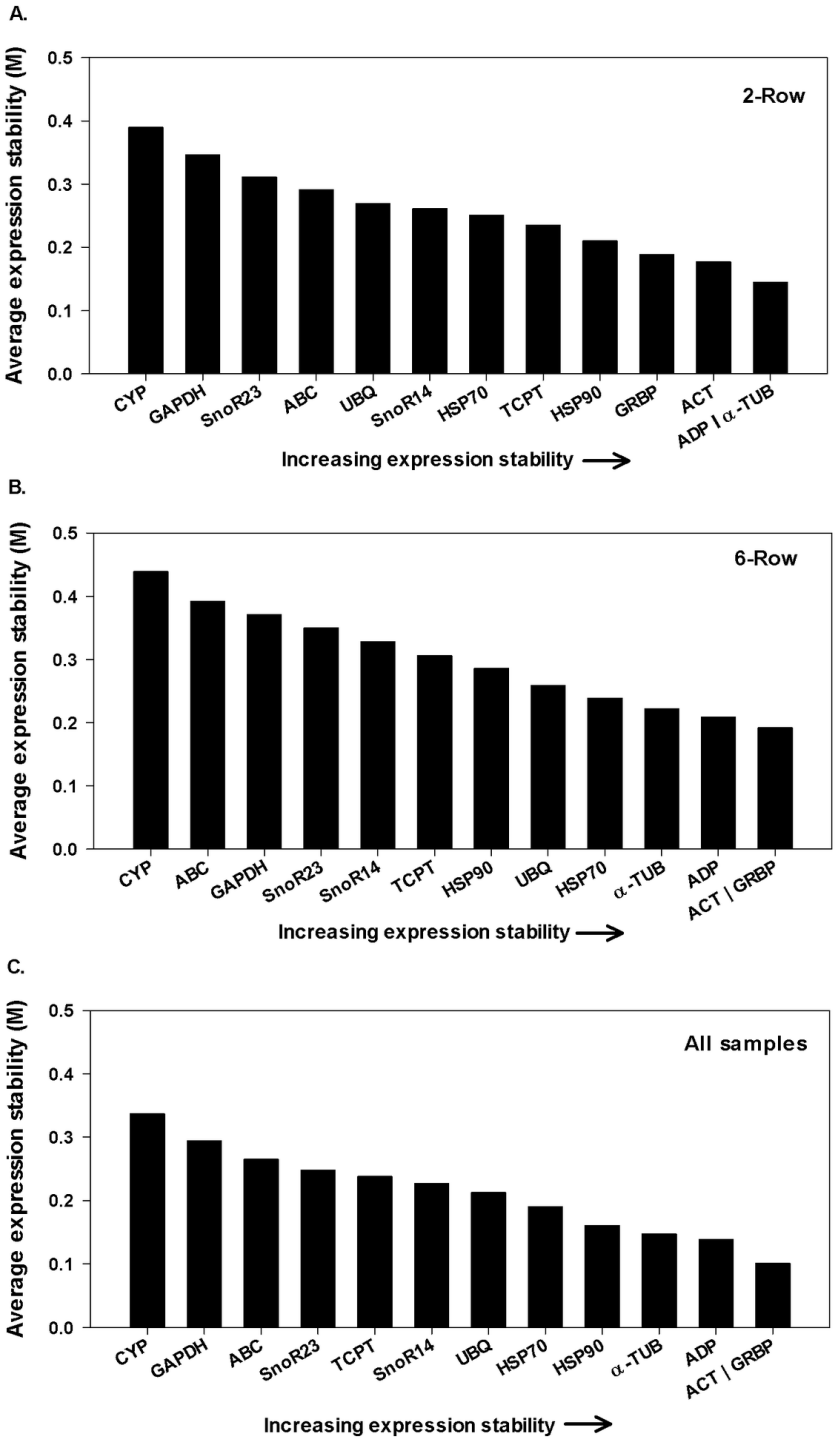
Expression stability of candidate reference genes analyzed by geNorm. Genes on the x-axis in order of increasing stability (y-axis M values) for (A) two-row barley, (B) six-row barley, and (C) all samples (two- and six-row combined).

All 13 reference genes were assessed for stability during micromalting in the two- and six-row samples independently and also combined (Fig 2). M values in both the two- and six-row barleys had min and max ranging between 0.4 to 0.15, which indicates that according to the stability standards considered by geNorm (M = <1.5), all thirteen putative reference genes are stable enough for experimental use in our sample set [24]. GeNorm found that the three most stable genes in the six-row malting barley Legacy were *Act* (M = 0.19), *GRBP* (M = 0.19) and *ADP* (M = 0.2). The least stable genes were *CYP* (M = 0.43) and *ABC* (M = 0.39). The ranking of reference genes in the two-row malting barley Conrad closely matched that suggested in the six-row analysis, with a few small changes. *CYP* (M = 0.39) was again the least stable with *GAPDH* the second least stable (M = 0.34). The three most stable genes were *ADP*, *α-TUB* and *ACT* (M = 0.145, 0.145, and 0.173 respectively).

When considering both barley types (two- and six-row together), geNorm provided a stability ranking which resembled that previously assigned to the six-row with *CYP*, *GAPDH*, *and ABC* being the least stable and *ACT* and *GRBP* being the most stable (Fig 2C).

#### NormFinder

**Fig 3:**
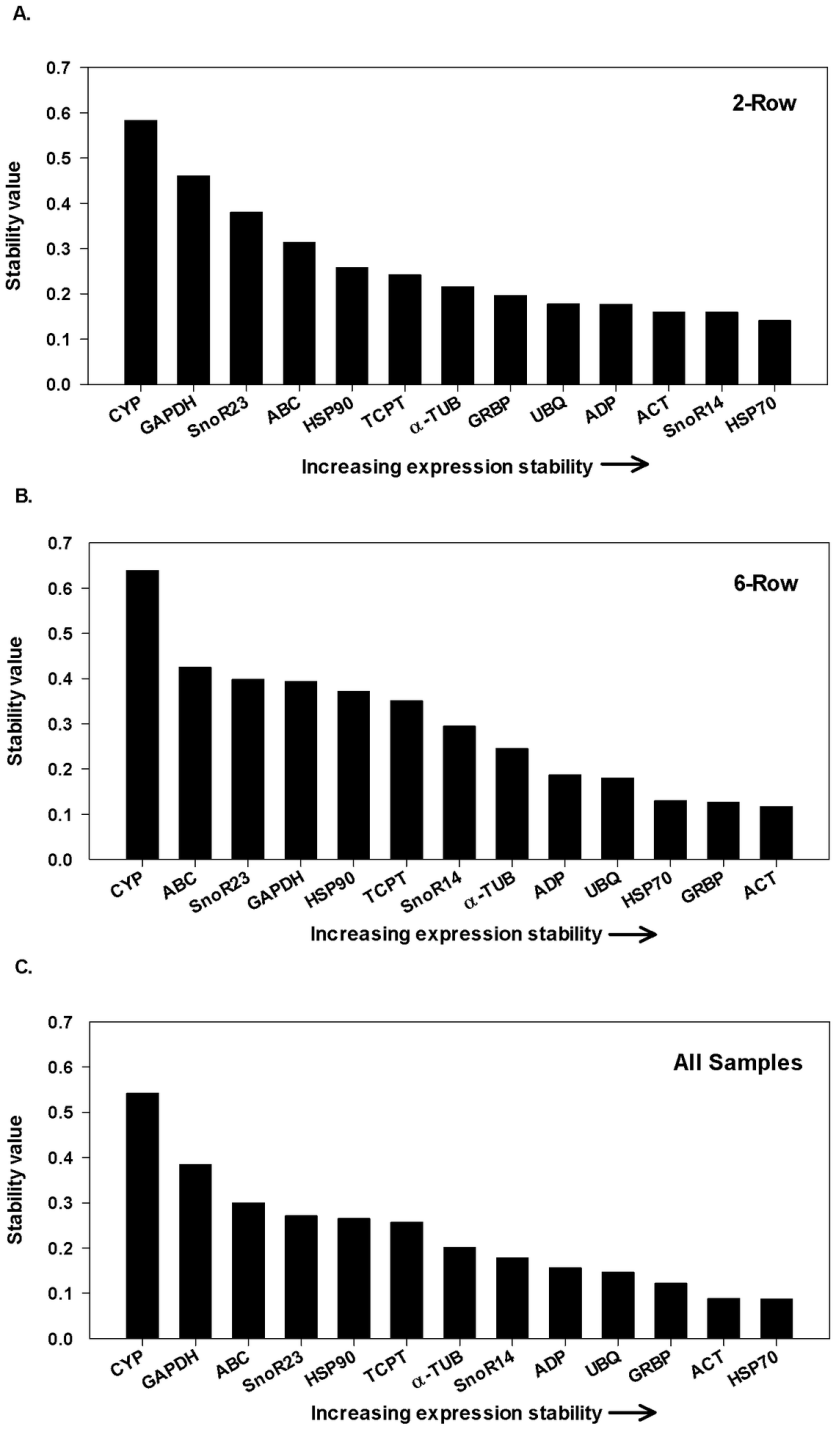
Gene expression stability values using NormFinder. Gene expression stability values from least stable (*left*) to most stable (*right*) for (A) two-row barley, (B) six-row barley, and (C) all samples (two- and six-row combined).

NormFinder, an Excel based program, measures the stability of each gene independently by considering, in a stepwise fashion, the inter-group and intra-group variation among samples using ANOVA [23]. Like geNorm, a larger value indicates a lower level of stability across treatments. *CYP* (0.583) and *GAPDH* (0.461) remain the two least stable genes in the two-row and *CYP* (0.639) and *ABC* (0.425) were the two least stable in the six-row; both results are consistent with those provided by geNorm (Figs 2B and 3B). Based on NormFinder, *ACT* (0.16), *SnoR14* (0.154), and *HSP70* (0.141) were the top three most stable in the two-row malting barley Conrad and *HSP70* (0.13), *GRBP* (0.126) and *ACT* (0.117) were the three most stable in the six-row malting barley Legacy. Across both row types, NormFinder ranked *CYP* and *GAPDH* as the two least stable and *GRPB*, *ACT* and *HSP70* as the three most stable (Fig 3C).

#### BestKeeper

BestKeeper takes a slightly different approach than geNorm and NormFinder by considering the variability of expression within samples, rather than pairwise comparisons, as calculated from the CV and standard deviation (SD) of the Ct thresholds within each of the candidate genes [25].

**Fig 4:**
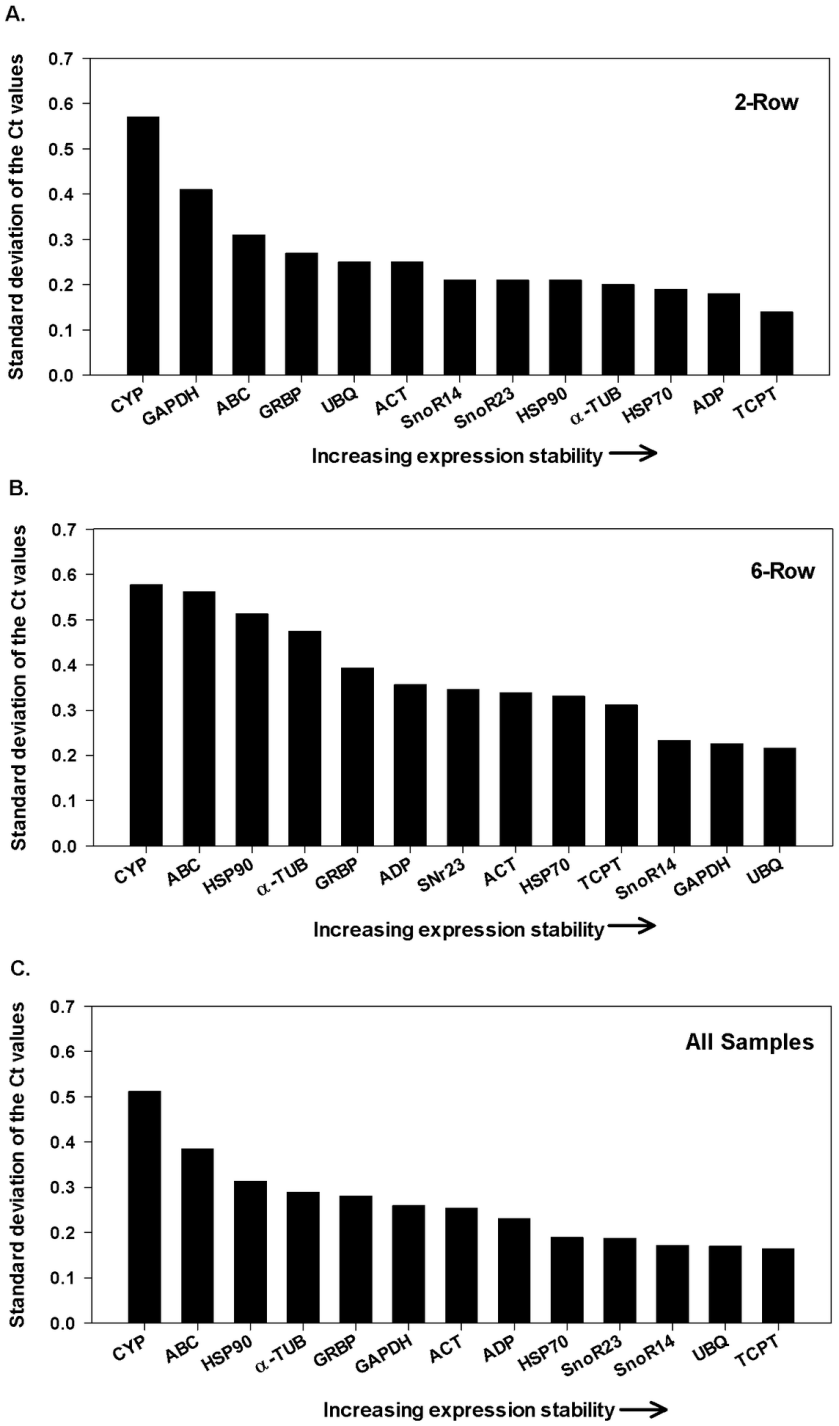
Gene expression stability values of 13 reference candidate genes using BestKeeper. Gene expression stability values of genes from the least stable (*left*) to the most stable (*right*) for (A) two-row barley, (B) six-row barley, and (C) all samples (two- and six-row combined).

While BestKeeper’s prediction of the least stable genes in both the two- and six-row micromalted barley samples were similar to those proposed by the other two programs [*CYP* (SD = 0.57) and *GAPDH* (SD = 0.41) in the two-row and *CYP* (SD = 0.57) and *ABC* (SD = .56) in the six-row], BestKeeper ranked *UBQ* (SD = 0.21) and *GAPDH* (0.23) as most stable in the six-row, and *TCTP* (SD = 0.14) and *ADP* (SD = 0.18) as the two most stable reference genes in the two-row, representing a disagreement in ranking between row types (*GAPDH* ranked #2 in six-row and #12 in two-row) as well as the other two programs that ranked these genes (*UBQ* and *GAPDH*) much lower (Fig 4). However, when data sets for two- and six-row were merged and analyzed together, *UBQ* and *TCPT* emerged as the two most stable genes and *GAPDH* dropped in ranking to the bottom five. *CYP* and *ABC* were once again ranked as the two least stable genes across both micromalted barley types.

Each of the three algorithms employed here provided a metric by which to make an informed choice as to which reference genes may provide the most stable controls among micromalted samples. In most cases, the rankings generated by all three programs resemble each other with Normfinder and geNorm generally sharing a higher degree of similarity than those generated by BestKeeper (Figs 2–4). The most striking example of a ranking that deviated from a consensus was observed in BestKeeper’s ranking of *GAPDH*. *GAPDH* was only ranked in the top three when BestKeeper was used and only for the six-row micromalted samples. In all other analyses *GAPDH* consistently ranked as one of the least stable (Figs 2–4). This phenomenon has been described in other reference gene evaluations where BestKeeper’s recommendations deviated from those provided by geNorm and Normfinder. Similar findings have been reported in barley [26,27], sorghum [22] and tobacco [21] where stability rankings were generated using the three algorithms and geNorm and Normfinder awarded the highest stability values to genes that were ranked lower by BestKeeper. The difference in results can be reconciled considering that each employs a different statistical approach to the rankings with BestKeeper relying heavily on the deviation of Ct values from the Ct mean as its main predictor of stability.

Normfinder and geNorm identified *CYP* as the least stable gene to use as normalizing targets in RT-qPCR experiments during micromalting in both two and six-row malting barley varieties. A similar panel of putative reference genes was evaluated in sorghum and using the same algorithms here, both *GAPDH* and *CYP* were determined to be the least stable [22,28]. The stability of *GAPDH* during germination has been examined in other species where it also ranked among the least stable reference genes. Tissues from germinating seeds and seedlings from *Plukenetia volubilis* were used to assess a set of putative reference primers and among those tested, *GAPDH* again was the least stable [29]. Furthermore, this same study reported *ACT* being the most stable reference gene for use in germinating seed of *P*. *volubilis*, corroborating our data and suggesting that *ACT* is one of the most stable genes tested during germination (malting is essentially controlled seed germination). While both *ACT* and *GAPDH* have been well documented in the literature as two of the more popular housekeeping genes for RT-qPCR normalization [2,28], our evaluation reveals the importance of testing each putative reference gene in your treatment samples rather than relying on the popularity by citation-count of a reference gene in the literature.

### Comprehensive weighting of gene stability using RefFinder

Each of the three described programs (geNorm, Normfinder, and BestKeeper) employed a slightly different statistical approach to rank the stability of the reference genes, and as a result, provided different but similar rankings [30]. To determine a consensus ranking of all the gene stabilities across sample time points from micromalted barleys, we used RefFinder which develops its ranking based on geometric means of ranked values provided from a suite of four programs including the three used earlier as well as a comparative ΔCt method [31].

Using each of the three programs, *CYP* and *GAPDH* consistently ranked as the two least stable in two-row malting barley Conrad, and as such, are reflected as the lowest ranked reference genes within the consensus output from RefSeq (Table 2). The consensus ranking for the most stable genes across the two-row micromalted samples were *HSP70*, *ADP*, and *α-TUB*, respectively. In the six-row micromalted samples, *CYP* was also the least stable gene, with *ABC* ranked as the next least stable, while *ACT*, *GRBP* and *UBQ* were ranked by RefFinder as the top three most stable genes.

**Table 2:** Stability rankings of candidate reference genes calculated by geNorm, NormFinder, and BestKeeper algorithms. Comprehensive ranking of candidate reference genes using RefFinder.

	Rank	geNorm	NormFinder	BestKeeper	RefFinder
**Two-Row Barley**	1	ADP | α-Tub	HSP70	TCPT	HSP70
	2		SnoR14	ADP	ADP
	3	ACT	ACT	HSP70	α-Tub
	4	GRBP	ADP	α-Tub	ACT
	5	HSP90	UBQ	SnoR14	SnoR14
	6	TCPT	GRBP	SnoR23	TCPT
	7	HSP70	α-Tub	HSP90	GRBP
	8	SnoR14	TCPT	UBQ	UBQ
	9	UBQ	HSP90	ACT	HSP90
	10	ABC	ABC	GRBP	SnoR23
	11	SnoR23	SnoR23	ABC	ABC
	12	GAPDH	GAPDH	GAPDH	GAPDH
	13	CYP	CYP	CYP	CYP
**Six-Row Barley**	1	ACT | GRBP	ACT	UBQ	ACT
	2		GRBP	GAPDH	GRBP
	3	ADP	HSP70	SnoR14	UBQ
	4	α-TUB	UBQ	TCPT	HSP70
	5	HSP70	ADP	HSP70	ADP
	6	UBQ	α-TUB	ACT	SnoR14
	7	HSP90	SnoR14	SnoR23	α-TUB
	8	TCPT	TCPT	ADP	TCPT
	9	SnoR14	HSP90	GRBP	GAPDH
	10	SnoR23	GAPDH	α-TUB	HSP90
	11	GAPDH	SnoR23	HSP90	SnoR23
	12	ABC	ABC	ABC	ABC
	13	CYP	CYP	CYP	CYP
**All samples**^a^	1	ACT | GRBP	HSP70	TCPT	ACT
	2		ACT	UBQ	HSP70
	3	ADP	GRBP	SnoR14	GRBP
	4	α-TUB	UBQ	SnoR23	UBQ
	5	HSP90	ADP	HSP70	ADP
	6	HSP70	SnoR14	ADP	TCPT
	7	UBQ	α-TUB	ACT	SnoR14
	8	SnoR14	TCPT	GAPDH	α-TUB
	9	TCPT	HSP90	GRBP	SnoR23
	10	SnoR23	SnoR23	α-TUB	HSP90
	11	ABC	ABC	HSP90	GAPDH
	12	GAPDH	GAPDH	ABC	ABC
	13	CYP	CYP	CYP	CYP

^a^ All samples: Two- and six-row samples combined.

A consensus ranking was also determined across both sample types using RefFinder and *ACT*, *GRBP*, *UBQ*, or *HSP70* were considered the most stable genes across both two- and six- row micromalted samples. *CYP*, *ABC*, *GAPDH* and *HSP90* received the lowest consensus stability ranking of the 13 putative reference genes examined (Table 2).

When the stability ranking of the two-row were compared with those from the six-row, by and large, the rankings resembled each other (Table 2, i.e. the top five and bottom five were similar albeit with some shuffling). Barley inflorescence architecture is governed by a small number of transcription factors that dictate the fertility of the lateral spikelets (*Vrs1*, *INT-C*, *VRS3*, and Vrs4) [8,32,33]. The genetic mutation that differentiates modern two- and six-row malting barleys is the *Vrs1* gene where the presence of the recessive *vrs1* allele causes the two-row phenotype [33]. Additionally, malting quality breeding guidelines for two- and six-row malting barley are similar highlighting the consistency required within the U.S. malting barley breeding programs [34]. All malting barley industries (e.g. beer brewers and distillers) require the barley to break dormancy quickly, germinate uniformly, and complete modification within four days [34]. Therefore, given the fact that both Legacy and Conrad have been bred for the same or very similar malting quality parameters it is unsurprising that there are such limited gene expression differences observed among the chosen putative housekeeping genes. Interestingly, despite the similarities in housekeeping gene expression the traditional assumptions of malting quality differences hold true. The six-row malting cultivar Legacy has higher color, higher protein levels (wort protein, S/T (%), and FAN), higher enzyme levels (DP, *α*-amylase activity), and higher β-glucan levels, while the two-row malting cultivar Conrad had higher kernel weight and more plump kernels (S1 Table). Malt extract was similar between the row types, which can be attributed to the tight requirement that malting barleys require within this parameter [34]. However, deviation of average Ct values is not uncommon, even when the sample types share a high degree of presumed similarity. For example, variation of observed average Ct values of reference genes deviated by as much as two-fold in cultivars of rice and four-fold among cultivars of petunia [35,36] again demonstrating the importance of stability testing under each sample type. It should be noted that while *GRBP* ranked in the top three reference genes by RefSeq across all samples, it should be used with caution given that the primers described here generate secondary amplicons in some samples.

### Optimal number of reference genes for RT-qPCR in micromalted samples

The choice of how many reference genes to employ can affect both the cost per sample of RT-qPCR analysis as well as the integrity and reliability of the data produced from such an experiment [28]. Since each reference gene included constitutes the amplification of the entire experimental sample set, the number of reference genes chosen can vastly affect overall number of reactions necessary to perform. Furthermore, it’s been documented in similar reference gene evaluations that the addition of more reference genes does not always increase the precision of the analysis, and, at times, may even hinder it. For example, two very stable reference genes may provide a better reference value alone than if used together with additional, but mediocre, reference genes [20].

Here, the analyses provided by geNorm, in addition to assigning a stability ranking, also proposes a minimal number of reference genes needed for faithful RT-qPCR analysis based on pairwise variation among the current stability-ranked genes being analyzed. geNorm assumes the two most stable reference genes to be used in a RT-qPCR experiment and determines whether the addition of the next most stable reference gene (V2/V3) into the experiment would provide additional statistical power. It continues to assess the power of adding additional putative reference genes (V3/V4….V12/13) from the ranked list until all genes have been included into the modeled experiment. geNorm recommends a minimal (or optimal) number of reference genes needed by providing a cut off value of 0.15 as the maximum pairwise variation allowable to perform RT-qPCR within a collection of reference genes [24].

**Fig 5:**
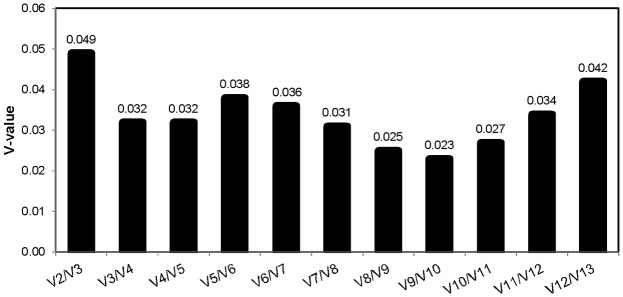
Determination of optimal number of reference genes for normalization by geNorm. Pairwise variation was calculated by geNorm to determine the minimum number of reference genes required for accurate normalization.

In both the two- and six-row micromalted samples, all combinations of putative reference genes had calculated pairwise variation values much lower than 0.15. The highest pairwise variation of genes used on either micromalted two- or six-row barley was observed in the V2/V3 analysis and were 0.050 and 0.061, respectively (Fig 5). Since both these values are well beneath the suggested cutoff of 0.15 provided by geNorm, the minimal number of reference genes needed is two. These data reflect reports from several reference gene primer evaluations where due to the high level of stability of all selected putative reference genes within a treatment, that the use of more than two reference primers does not necessarily always increase the precision of the analysis [15,22,27,37]. The high degree of stability among all the genes tested here may reflect the nature of the micromalting process whereby the extent of stress is minimal or subdued compared to those induced by more pronounced stress sources such as drought. Therefore the transcription of the selected reference genes has a higher incidence of consistency [15]. Interestingly, all thirteen genes remained stable enough to be used as reference genes despite the ten total biological replications (Five from two-row Conrad and five from six-row Legacy) being grown over four years in four different geographic locations (S1 Table). Additionally, both two- and six-row malting barley varieties have been bred to easily break dormancy and modify (i.e. germinate) completely in as little time as possible indicating putative housekeeping genes in both row types would behave similarly [34]. However, more likely, the high scoring of all genes tested here reflects the selective choice of reference genes from previous reports that have already vetted and thus discarded putative reference genes that do not reflect an acceptable level of stability across varied treatments and sample types.

## Conclusion

This report represents the first evaluation of reference primers across micromalted samples of both two- and six-row barleys. The suite of primers described here represent a survey of both traditional housekeeping genes as well as more contemporary targets such as small nucleolar RNAs. We used three of the most popular algorithms (geNorm, NormFinder, and BestKeeper) to generate stability values necessary for ranking our putative reference genes. The rankings supplied by these algorithms were similar but deviated to some degree from each other due to each having unique approaches to determine overall stability. We therefore used RefSeq to provide a consensus ranking from all gene stability values from four difference ranking algorithms, however it should be noted that each of the computational methods we used produced comparable overall rankings and therefore given BestKeeper is available at no cost, may represent the best choice for routine analysis reference primer stability. We found that all genes assayed for use as a reference primer in RT-qPCR experiments generally performed well, each demonstrating a consistent level of stable expression across micromalt stages and samples type. That said, we were able to identify *Act* and *HSP70* as the two most stable genes of those while *ABC* and *CYP* were the least stable. Our data suggest the use of only two reference genes to be sufficient for faithful normalization under these experimental conditions.

## Supporting information

S1 Fig Malting process at the Cereal Crops Research Unit. Barley in Steeping Cans (SC) are imbibed for 36 hours in the Steep Tank (ST). Imbibed barley is transferred to a Germination Chamber (GC) where they continue to germinate for five days. DoG 1–5 = Days of Germination.
(PDF)

S2 Fig Amplicon data from 13 reference gene pairs. A. Melt curves from each reference gene amplicon. B. Polyacrylamide gel electrophoresis of PCR products generated from reference gene primers.
(DOCX)

S1 Table Malting quality data for two malting varieties used in this study: Conrad (two-row) and Legacy (six-row). (DOCX)

## References

[1] Huggett J, Dheda K, Bustin S, Zumla A. Real-time RT-PCR normalisation; strategies and considerations. Genes Immun. 2005 6;6(4):279–84. doi: 10.1038/sj.gene.6364190 15815687 10.1038/sj.gene.6364190

[2] Thellin O, Zorzi W, Lakaye B, De Borman B, Coumans B, Hennen G, et al Housekeeping genes as internal standards: use and limits. J Biotechnol. 1999 10 8;75(2–3):291–5. 10617337 10.1016/s0168-1656(99)00163-7

[3] Remans T, Keunen E, Bex GJ, Smeets K, Vangronsveld J, Cuypers A. Reliable gene expression analysis by reverse transcription-quantitative PCR: reporting and minimizing the uncertainty in data accuracy. Plant Cell. 2014 10;26(10):3829–37. doi: 10.1105/tpc.114.130641 25361954 10.1105/tpc.114.130641 PMC4247583

[4] Gutierrez L, Mauriat M, Guénin S, Pelloux J, Lefebvre J-F, Louvet R, et al The lack of a systematic validation of reference genes: a serious pitfall undervalued in reverse transcription-polymerase chain reaction (RT-PCR) analysis in plants. Plant Biotechnol J. 2008 8;6(6):609–18. doi: 10.1111/j.1467-7652.2008.00346.x 18433420 10.1111/j.1467-7652.2008.00346.x

[5] Bustin SA, Benes V, Garson JA, Hellemans J, Huggett J, Kubista M, et al The MIQE guidelines: minimum information for publication of quantitative real-time PCR experiments. Vol. 55, Clinical chemistry. 2009 pp. 611–22. 10.1373/clinchem.2008.112797 19246619

[6] Bustin SA. Why the need for qPCR publication guidelines?—The case for MIQE. Methods. 2010 4;50(4):217–26. doi: 10.1016/j.ymeth.2009.12.006 20025972 10.1016/j.ymeth.2009.12.006

[7] American Malting Barley Association, Inc., AMBA. The Nationa Barley Improvement Committee 2017 Congressional Packet [Internet]. Milwaukee; [cited 2017 Sep 25]. http://ambainc.org/media/AMBA_PDFs/NBIC/2017_NBIC_Packet.pdf

[8] Koppolu R, Anwar N, Sakuma S, Tagiri A, Lundqvist U, Pourkheirandish M, et al Six-rowed spike4 (Vrs4) controls spikelet determinacy and row-type in barley. Proc Natl Acad Sci USA. 2013 8 6;110(32):13198–203. doi: 10.1073/pnas.1221950110 23878219 10.1073/pnas.1221950110 PMC3740847

[9] Schwarz P, Horsley R. A comparison of North American two-row and six-row malting barley. Brewing Techniques. 1996 11 30;48–55.

[10] Briggs DE. Malting In: Barley. London; 1978 pp. 526–59.

[11] Schwarz. History of malting barley in the United States, 1600—present. Master Brewers Association of America Technical Quarterly. 2012;49(3)103–107.

[12] American Malting Barley Association, Inc., AMBA. Malting Barley in North America [Internet]. [cited 2017 Sep 25]. http://ambainc.org/media/AMBA_PDFs/Member/Presentations/Heisel_MBAA_MN_11Sept2015.pdf

[13] Vinje MA, Duke SH, Henson CA. Comparison of factors involved in starch degradation in barley germination under laboratory and malting conditions. J Am Soc Brew Chem. 2015;73(2):195–205.

[14] Vinje MA, Willis DK, Duke SH, Henson CA. Differential RNA expression of *Bmy1* during barley seed development and the association with β-amylase accumulation, activity, and total protein. Plant Physiol Biochem. 2011 1;49(1):39–45. doi: 10.1016/j.plaphy.2010.09.019 20974538 10.1016/j.plaphy.2010.09.019

[15] Ferdous J, Li Y, Reid N, Langridge P, Shi B-J, Tricker PJ. Identification of reference genes for quantitative expression analysis of microRNAs and mRNAs in barley under various stress conditions. PLOS One. 2015;10(3):e0118503 doi: 10.1371/journal.pone.0118503 25793505 10.1371/journal.pone.0118503 PMC4368757

[16] Huang B, Hennen-Bierwagen TA, Myers AM. Functions of multiple genes encoding ADP-glucose pyrophosphorylase subunits in maize endosperm, embryo, and leaf. Plant Physiol. 2014 2;164(2):596–611. doi: 10.1104/pp.113.231605 24381067 10.1104/pp.113.231605 PMC3912092

[17] Ovesná J, Kučera L, Vaculová K, Štrymplová K, Svobodová I, Milella L. Validation of the β-amy1 transcription profiling assay and selection of reference genes suited for a RT-qPCR assay in developing barley caryopsis. Schönbach C, editor. PLOS One. 2012;7(7):e41886 doi: 10.1371/journal.pone.0041886 22860024 10.1371/journal.pone.0041886 PMC3409215

[18] Kaczmarczyk A, Bowra S, Elek Z, Vincze E. Quantitative RT-PCR based platform for rapid quantification of the transcripts of highly homologous multigene families and their members during grain development. BMC Plant Biol. 2012;12(1):184. 23043496 10.1186/1471-2229-12-184 PMC3492166

[19] Livak KJ, Schmittgen TD. Analysis of relative gene expression data using real-time quantitative PCR and the 2(-Delta Delta Ct) Method. Methods. 2001 12;25(4):402–8. doi: 10.1006/meth.2001.1262 11846609 10.1006/meth.2001.1262

[20] Chervoneva I, Li Y, Schulz S, Croker S, Wilson C, Waldman SA, et al Selection of optimal reference genes for normalization in quantitative RT-PCR. BMC Bioinformatics. 2010;11(1):253. 20470420 10.1186/1471-2105-11-253 PMC2889935

[21] Liu D, Shi L, Han C, Yu J, Li D, Zhang Y. Validation of reference genes for gene expression studies in virus-infected *Nicotiana benthamiana* using quantitative real-time PCR. PLOS One. 2012;7(9):e46451 doi: 10.1371/journal.pone.0046451 23029521 10.1371/journal.pone.0046451 PMC3460881

[22] Sudhakar Reddy P, Srinivas Reddy D, Sivasakthi K, Bhatnagar-Mathur P, Vadez V, Sharma KK. Evaluation of sorghum [*Sorghum bicolor* (L.)] reference genes in various tissues and under abiotic stress conditions for quantitative real-time PCR data normalization. Front Plant Sci. 2016;7:529 doi: 10.3389/fpls.2016.00529 27200008 10.3389/fpls.2016.00529 PMC4843019

[23] Kozera B, Rapacz M. Reference genes in real-time PCR. J Appl Genet. 2013;54(4):391–406. doi: 10.1007/s13353-013-0173-x 24078518 10.1007/s13353-013-0173-x PMC3825189

[24] Vandesompele J, De Preter K, Pattyn F, Poppe B, Van Roy N, De Paepe A, et al Accurate normalization of real-time quantitative RT-PCR data by geometric averaging of multiple internal control genes. Genome Biol. 2002 6 18;3(7):1–12. 10.1186/gb-2002-3-7-research0034 PMC126239 12184808

[25] Pfaffl MW, Tichopad A, Prgomet C, Neuvians TP. Determination of stable housekeeping genes, differentially regulated target genes and sample integrity: BestKeeper—Excel-based tool using pair-wise correlations. Biotechnol Lett. 2004 3;26(6):509–15. 15127793 10.1023/b:bile.0000019559.84305.47

[26] Rapacz M, Stępień A, Skorupa K. Internal standards for quantitative RT-PCR studies of gene expression under drought treatment in barley (*Hordeum vulgare* L.): the effects of developmental stage and leaf age. Acta Physiol Plant. 2012;34(5):1723–33.

[27] Hua W, Zhu J, Shang Y, Wang J, Jia Q, Yang J. Identification of suitable reference genes for barley gene expression under abiotic stresses and hormonal treatments. Plant Mol Biol Rep. 2014;33(4):1002–12.

[28] Dheda K, Huggett JF, Chang JS, Kim LU, Bustin SA, Johnson MA, et al The implications of using an inappropriate reference gene for real-time reverse transcription PCR data normalization. Anal Biochem. 2005 9;344(1):141–3. doi: 10.1016/j.ab.2005.05.022 16054107 10.1016/j.ab.2005.05.022

[29] Niu L, Tao Y-B, Chen M-S, Fu Q, Li C, Dong Y, et al Selection of reliable reference genes for gene expression studies of a promising oilseed crop, *Plukenetia volubilis*, by real-time quantitative PCR. IJMS. 2015 6 3;16(6):12513–30. doi: 10.3390/ijms160612513 26047338 10.3390/ijms160612513 PMC4490458

[30] De Spiegelaere W, Dern-Wieloch J, Weigel R, Schumacher V, Schorle H, Nettersheim D, et al Reference gene validation for RT-qPCR, a note on different available software packages. PLOS One. 2015;10(3):e0122515 doi: 10.1371/journal.pone.0122515 25825906 10.1371/journal.pone.0122515 PMC4380439

[31] Taylor CM, Jost R, Erskine W, Nelson MN. Identifying stable reference genes for qRT-PCR normalisation in gene expression studies of narrow-leafed lupin (*Lupinus angustifolius* L.). PLOS One. 2016;11(2):e0148300 doi: 10.1371/journal.pone.0148300 26872362 10.1371/journal.pone.0148300 PMC4752343

[32] van Esse GW, Walla A, Finke A, Koornneef M, Pecinka A, Korff von M. Six-rowed spike3 (VRS3) is a histone demethylase that controls lateral spikelet development in barley. Plant Physiol. 2017 8;174(4):2397–408. doi: 10.1104/pp.17.00108 28655778 10.1104/pp.17.00108 PMC5543938

[33] Komatsuda T, Pourkheirandish M, He C, Azhaguvel P, Kanamori H, Perovic D, et al Six-rowed barley originated from a mutation in a homeodomain-leucine zipper I-class homeobox gene. P Natk Acad Sci USA. 2007 1 23;104(4):1424–9. 10.1073/pnas.0608580104 PMC1783110 17220272

[34] American Malting Barley Association, Inc., AMBA. Malting Barley Breeding Guidelines Ideal Commercial Malt Criteria [Internet]. Milwaukee; [cited 2017 Sep 25]. http://ambainc.org/media/AMBA_PDFs/Pubs/Malting_Barley_Breeding_Guidelines_April_2017.pdf

[35] Kim B-R, Nam H-Y, Kim S-U, Kim S-I, Chang Y-J. Normalization of reverse transcription quantitative-PCR with housekeeping genes in rice. Biotechnol Lett. 2003;25:1869–72. 14677714 10.1023/a:1026298032009

[36] Mallona I, Lischewski S, Weiss J, Hause B, Egea-Cortines M. Validation of reference genes for quantitative real-time PCR during leaf and flower development in Petunia hybrida. BMC Plant Biol. 2010 1 7;10:4 doi: 10.1186/1471-2229-10-4 20056000 10.1186/1471-2229-10-4 PMC2827423

[37] Wu J, Zhang H, Liu L, Li W, Wei Y, Shi S. Validation of reference genes forRT-qPCR studies of gene expression in preharvest and postharvest longan fruits under different experimental conditions. Front Plant Sci. 2016;7:780 doi: 10.3389/fpls.2016.00780 27375640 10.3389/fpls.2016.00780 PMC4891570

