# Supplementary material for: Evaluation and selection of internal reference genes from two- and six-row U.S. malting barley varieties throughout micromalting for use in RT-qPCR
Source: PLoS One. 2018 May 8;13(5):e0196966. doi: 10.1371/journal.pone.0196966 (PMC5940201; doi:10.1371/journal.pone.0196966)
Supplement: S1 Fig — Barley in Steeping Cans (SC) are imbibed for 36 hours in the Steep Tank (ST). Imbibed barley is transferred to a Germination Chamber (GC) where they continue to germinate for five days. DoG 1–5 = Days of Germination. (PDF) [file pone.0196966.s001.pdf]

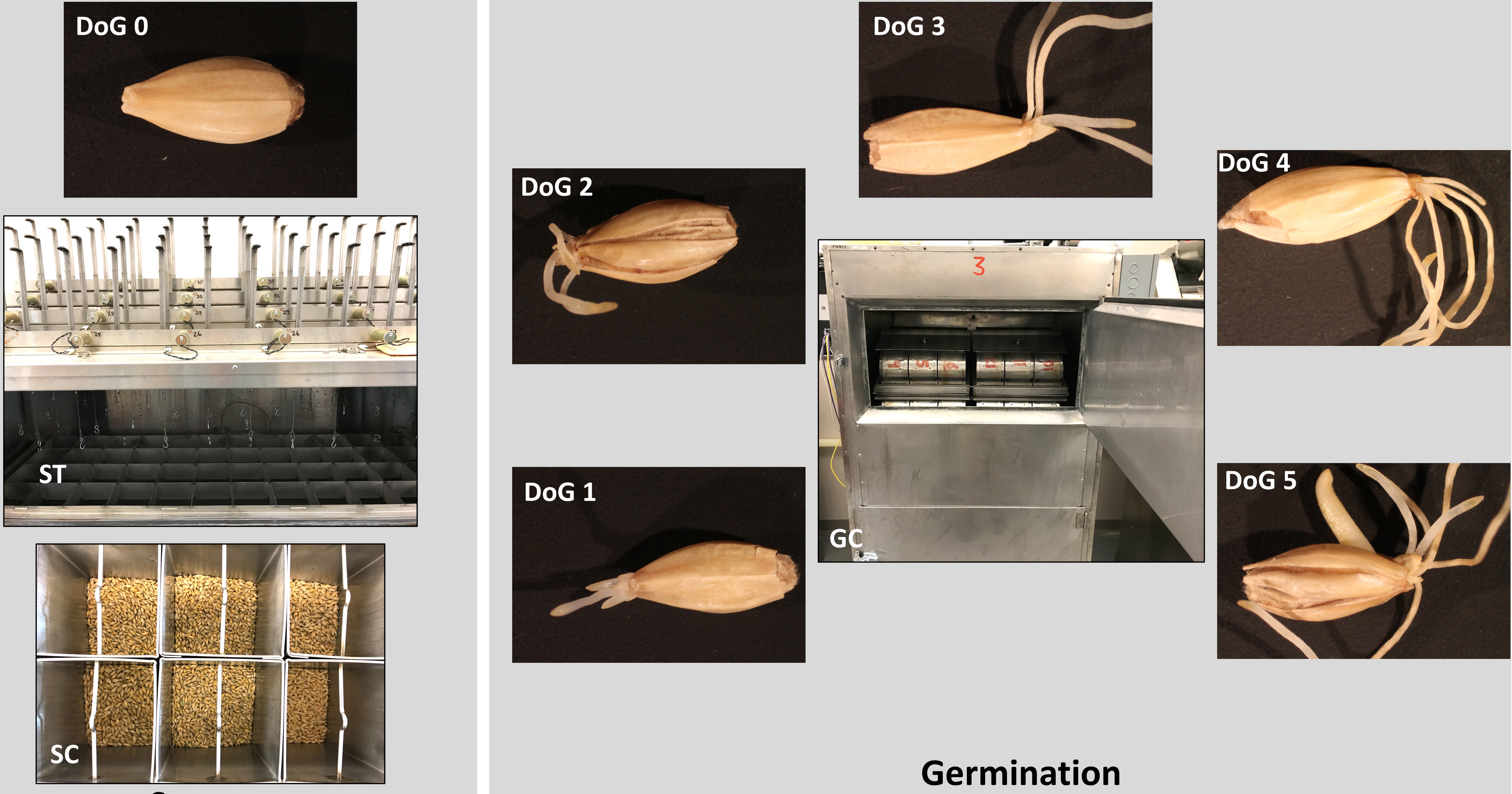

S1 Figure. Malting Process at CCRU. Barley in Steeping Cans (SC) are imbibed for 36 hours in the Steep Tank (ST). Imbibed barley is transferred to a Germination Chamber (GC) where they continue to germinate for 5 days. DoG 1-5 = Days of Germination.
