# Supplementary material for: Evaluation and selection of internal reference genes from two- and six-row U.S. malting barley varieties throughout micromalting for use in RT-qPCR
Source: PLoS One. 2018 May 8;13(5):e0196966. doi: 10.1371/journal.pone.0196966 (PMC5940201; doi:10.1371/journal.pone.0196966)
Supplement: S2 Fig — A. Melt curves from each reference gene amplicon. B. Polyacrylamide gel electrophoresis of PCR products generated from reference gene primers. (DOCX) [file pone.0196966.s002.docx]

**S2A Fig. Melt curves from each reference gene amplicon.**


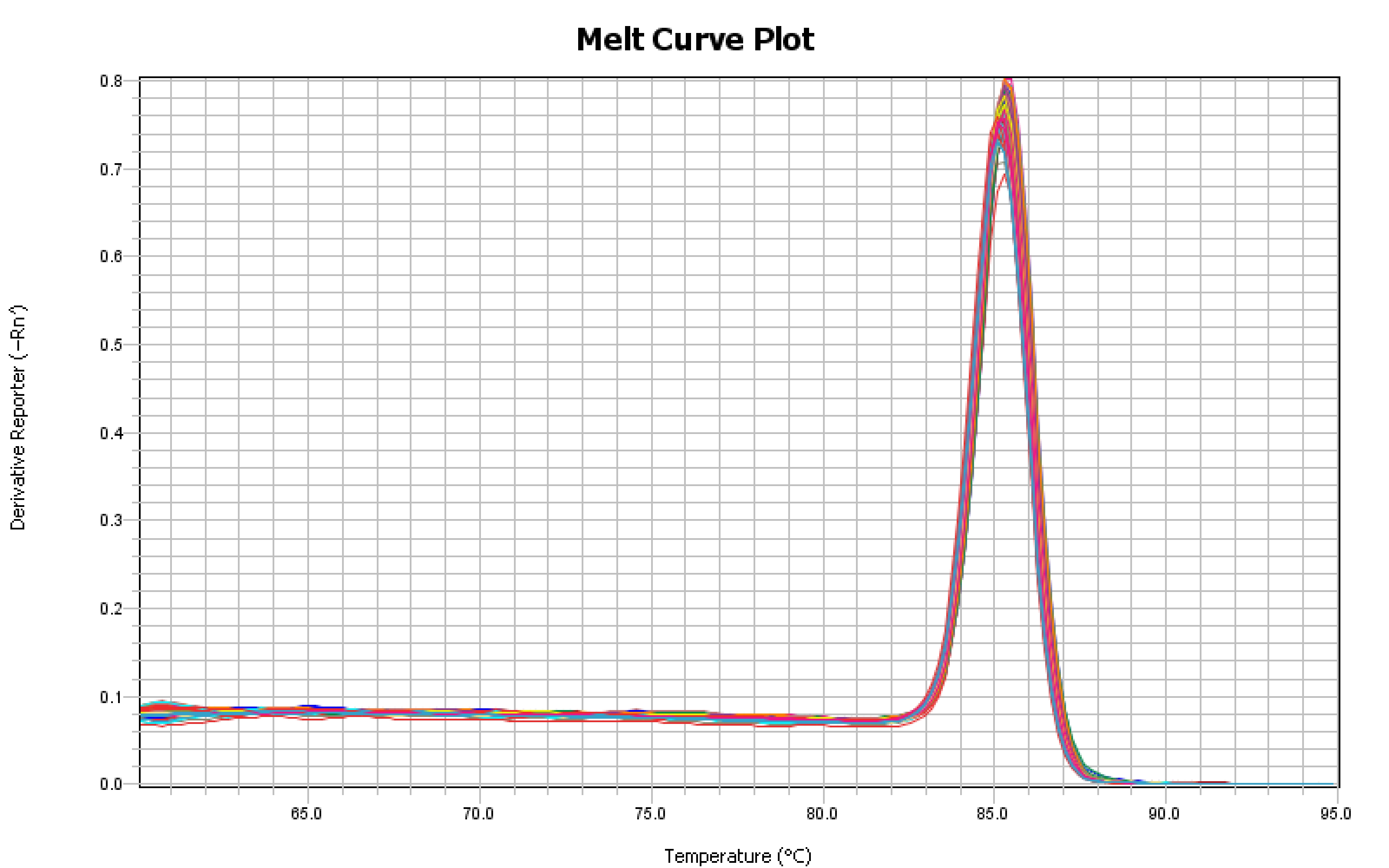

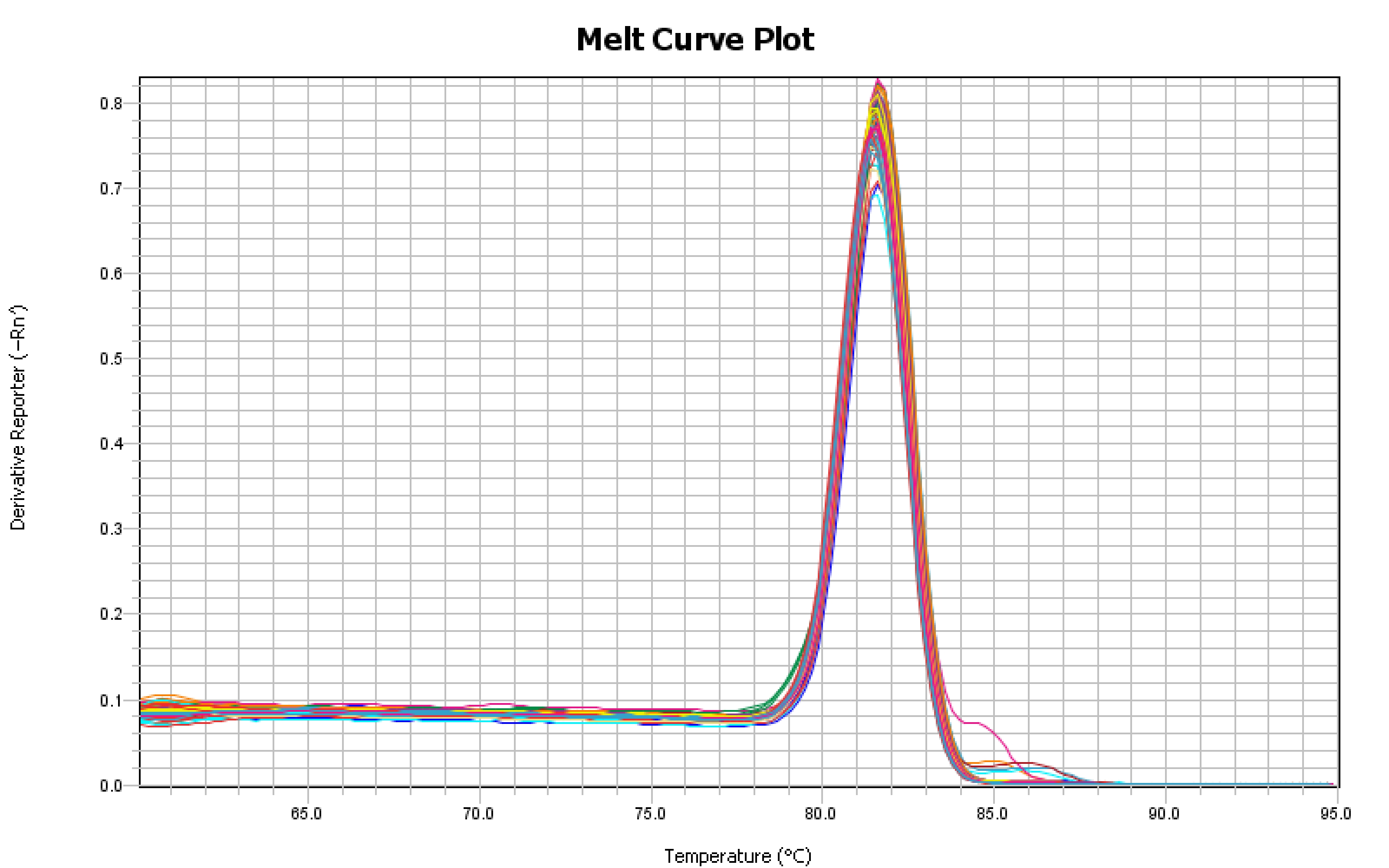


**ABC**

**ACT**


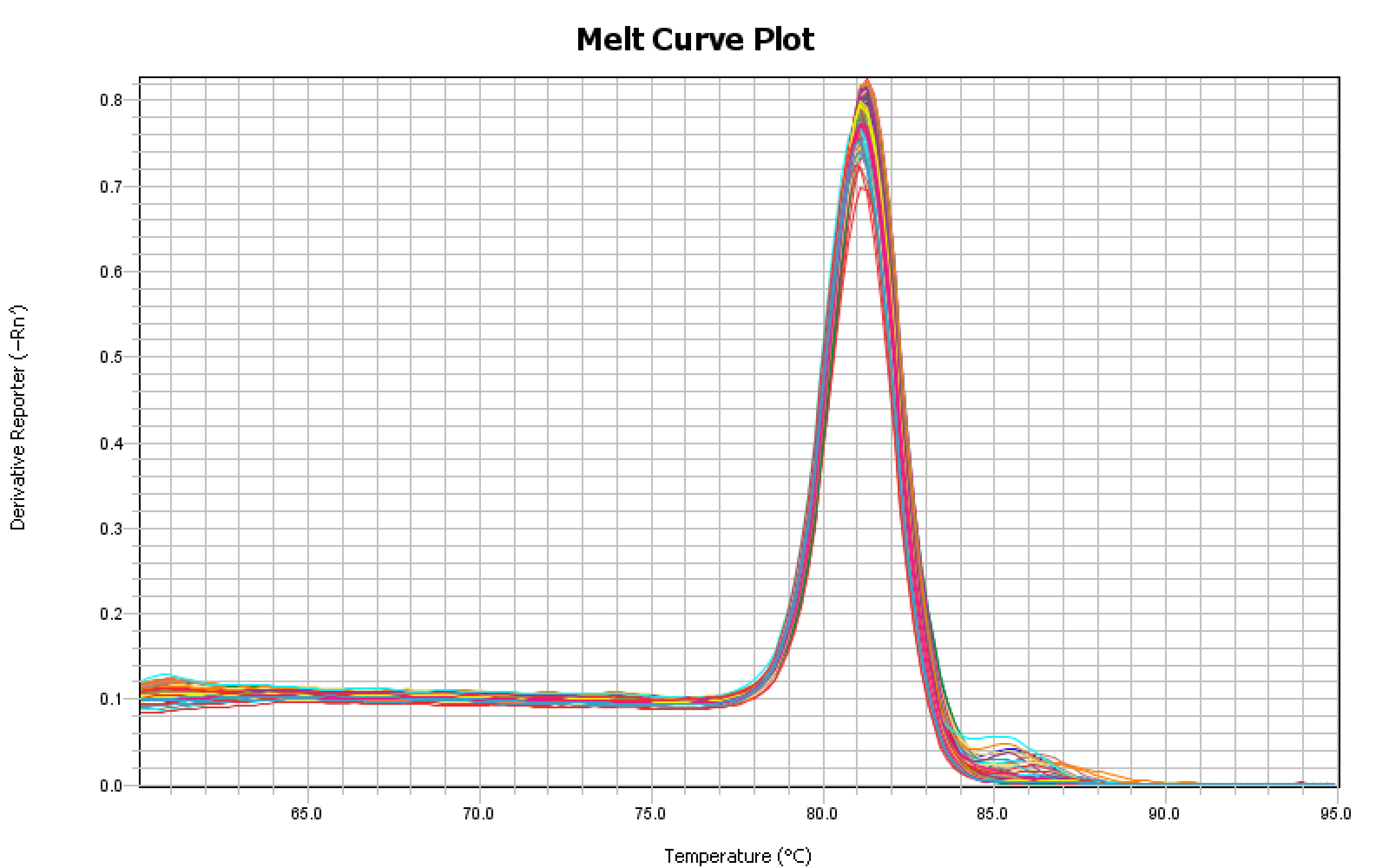

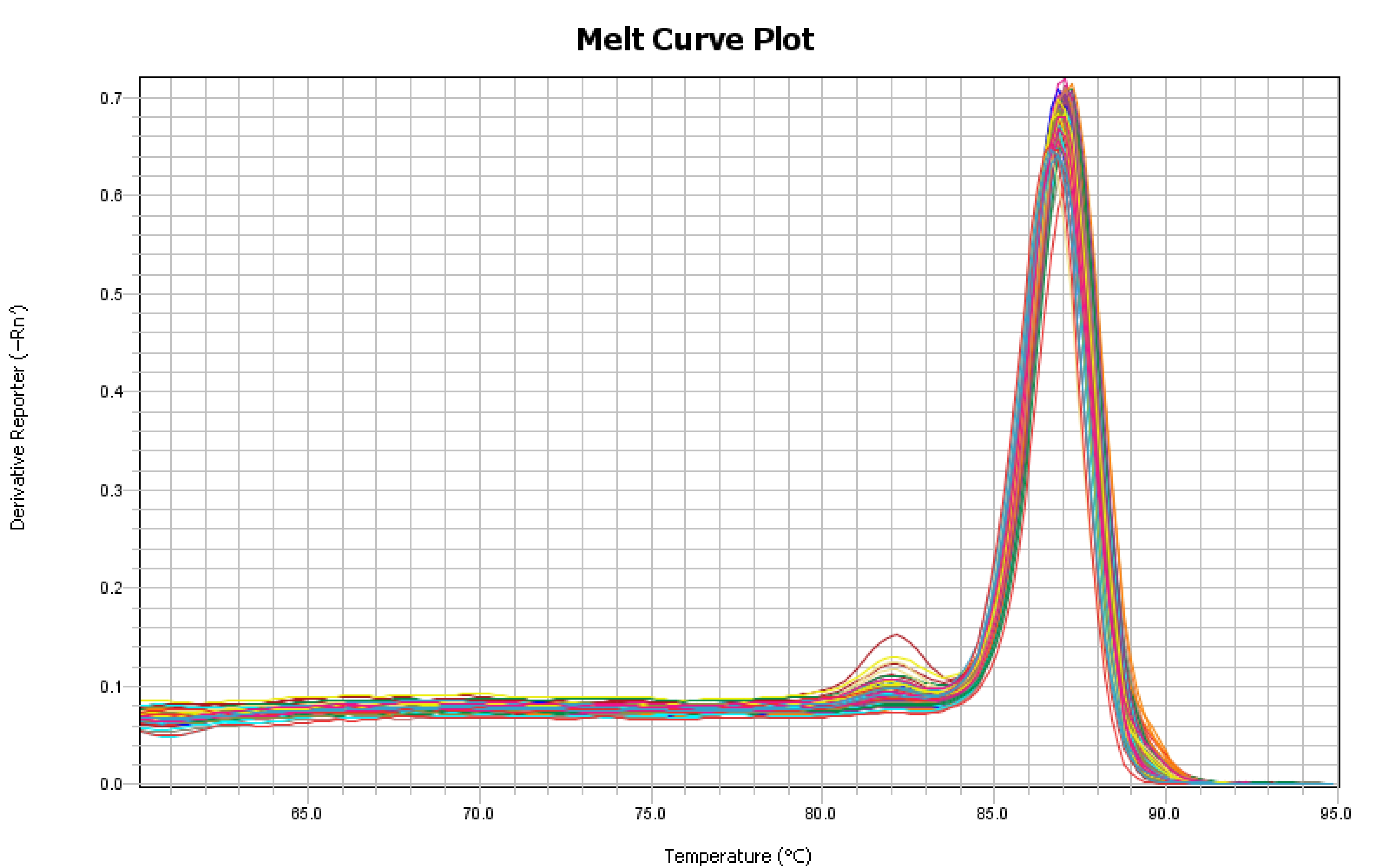


**ADP**

**α-TUB**


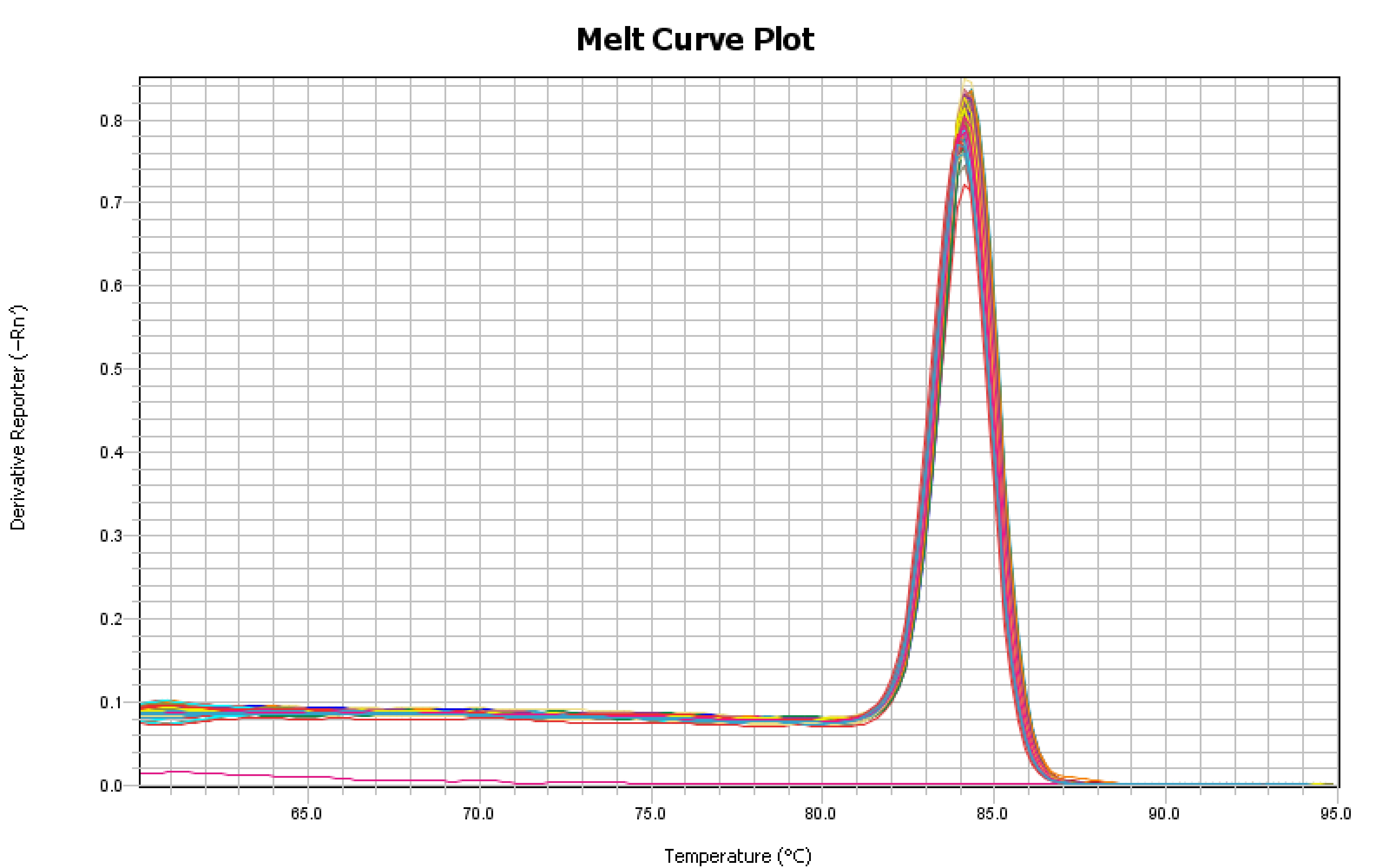

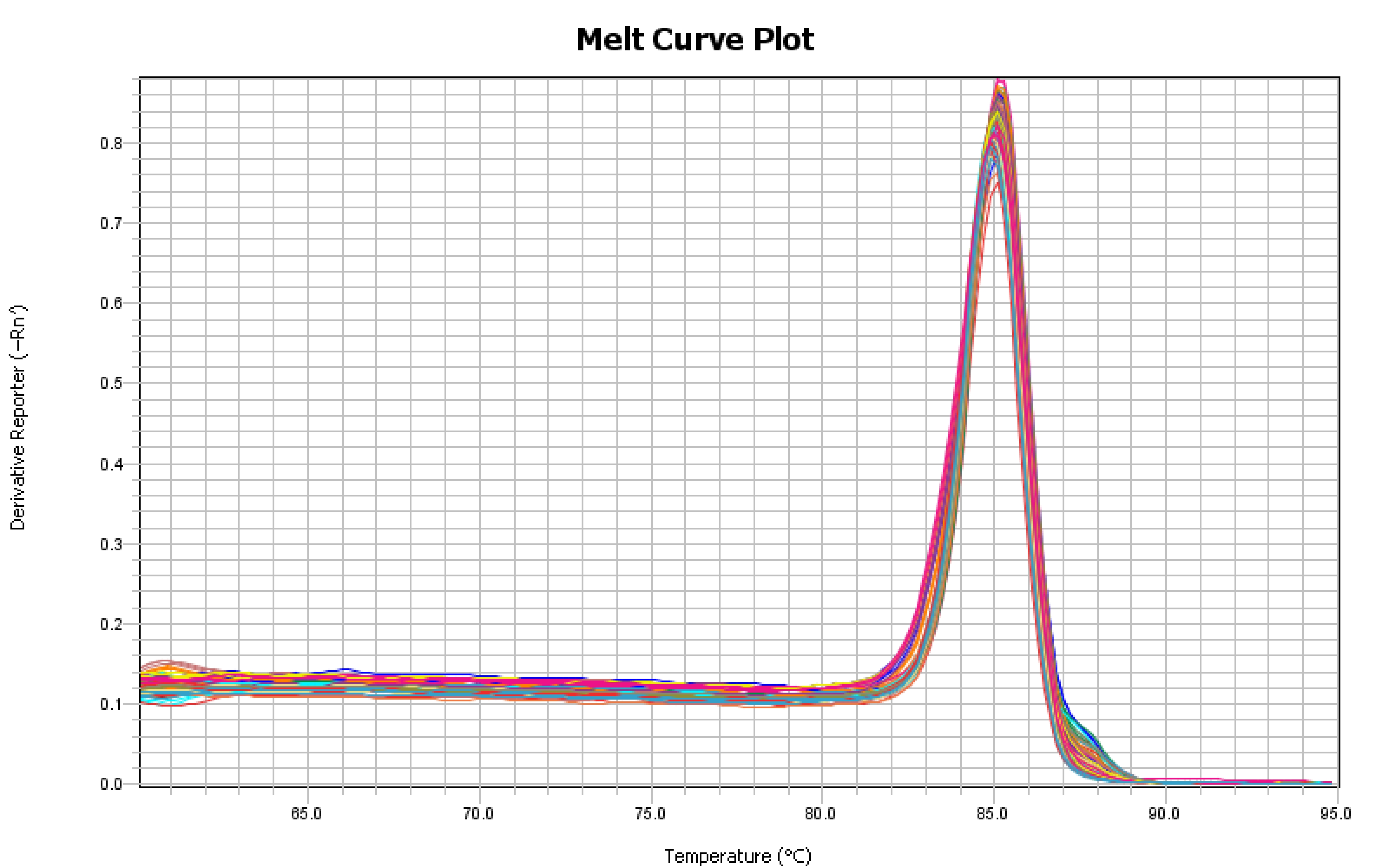


**GAPDH**

**CYP**


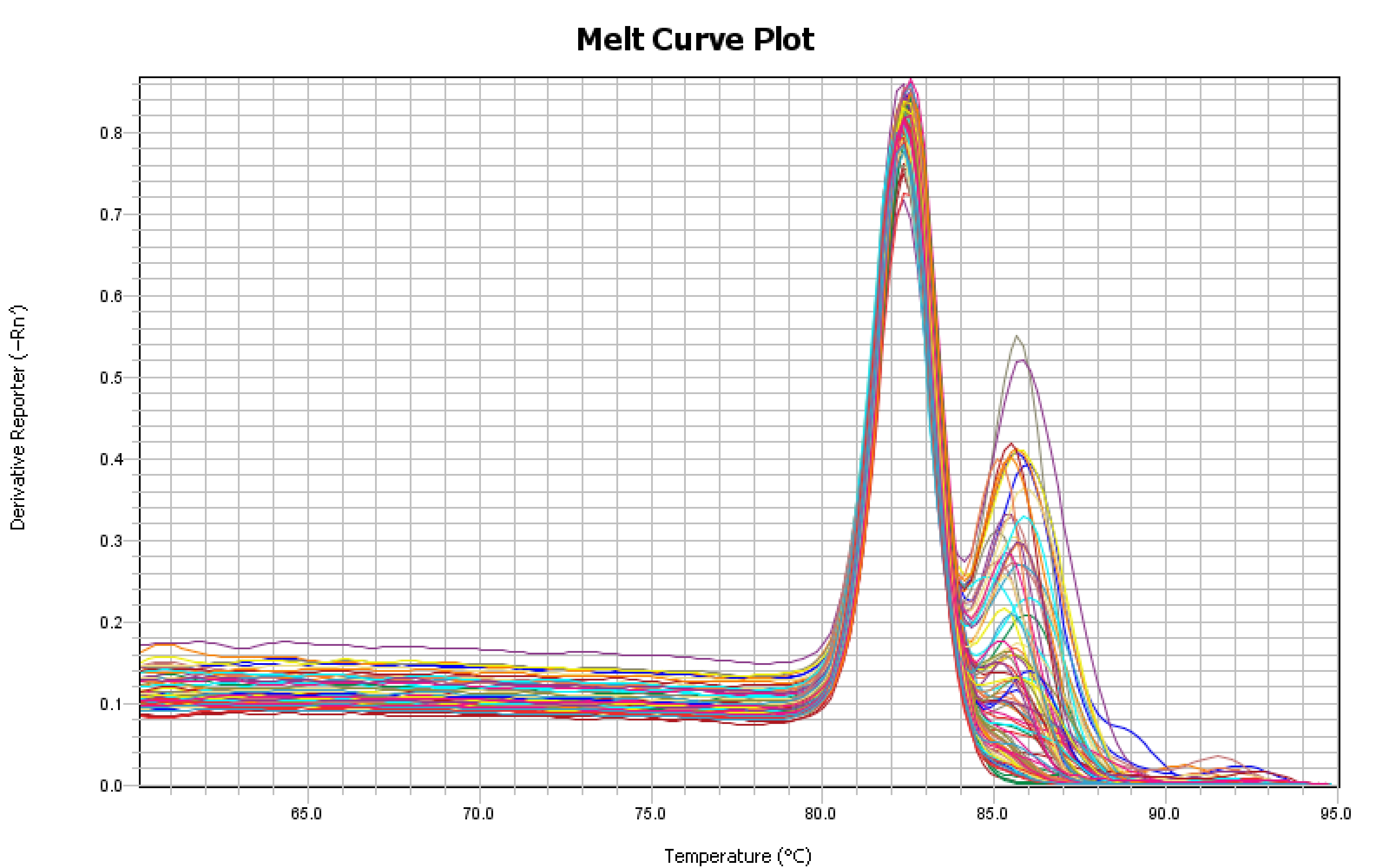

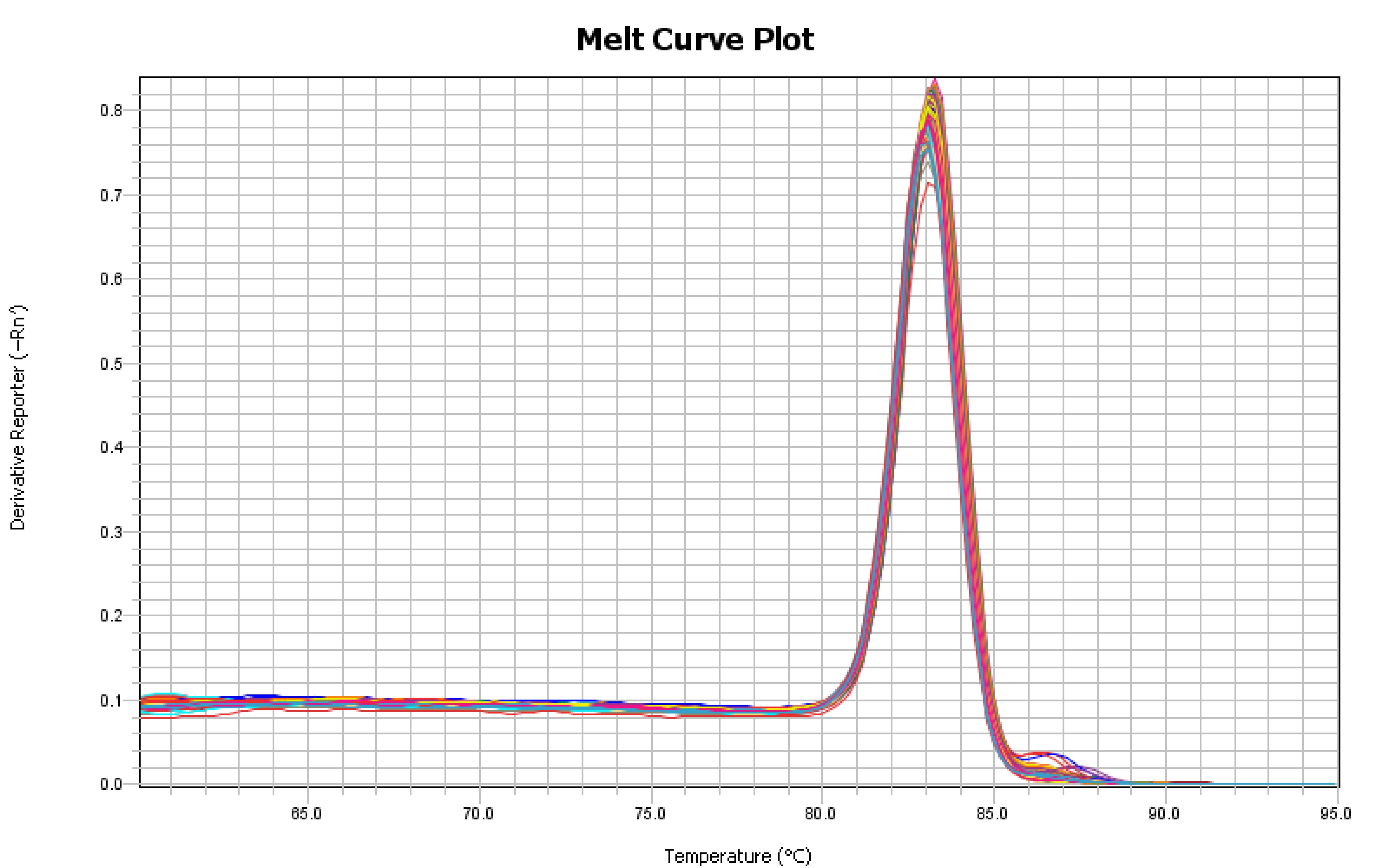


**HSP70**

**GRBP**


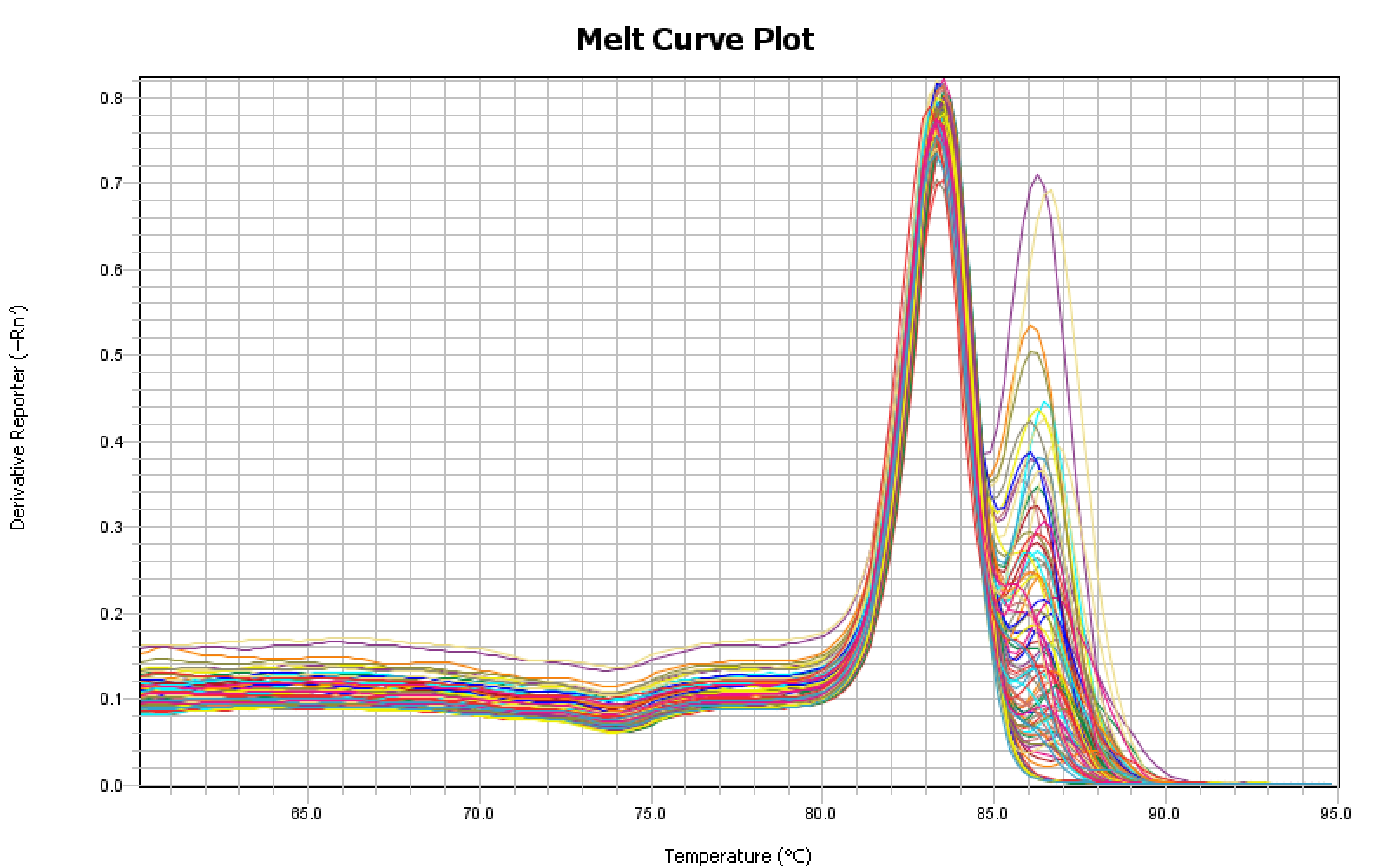

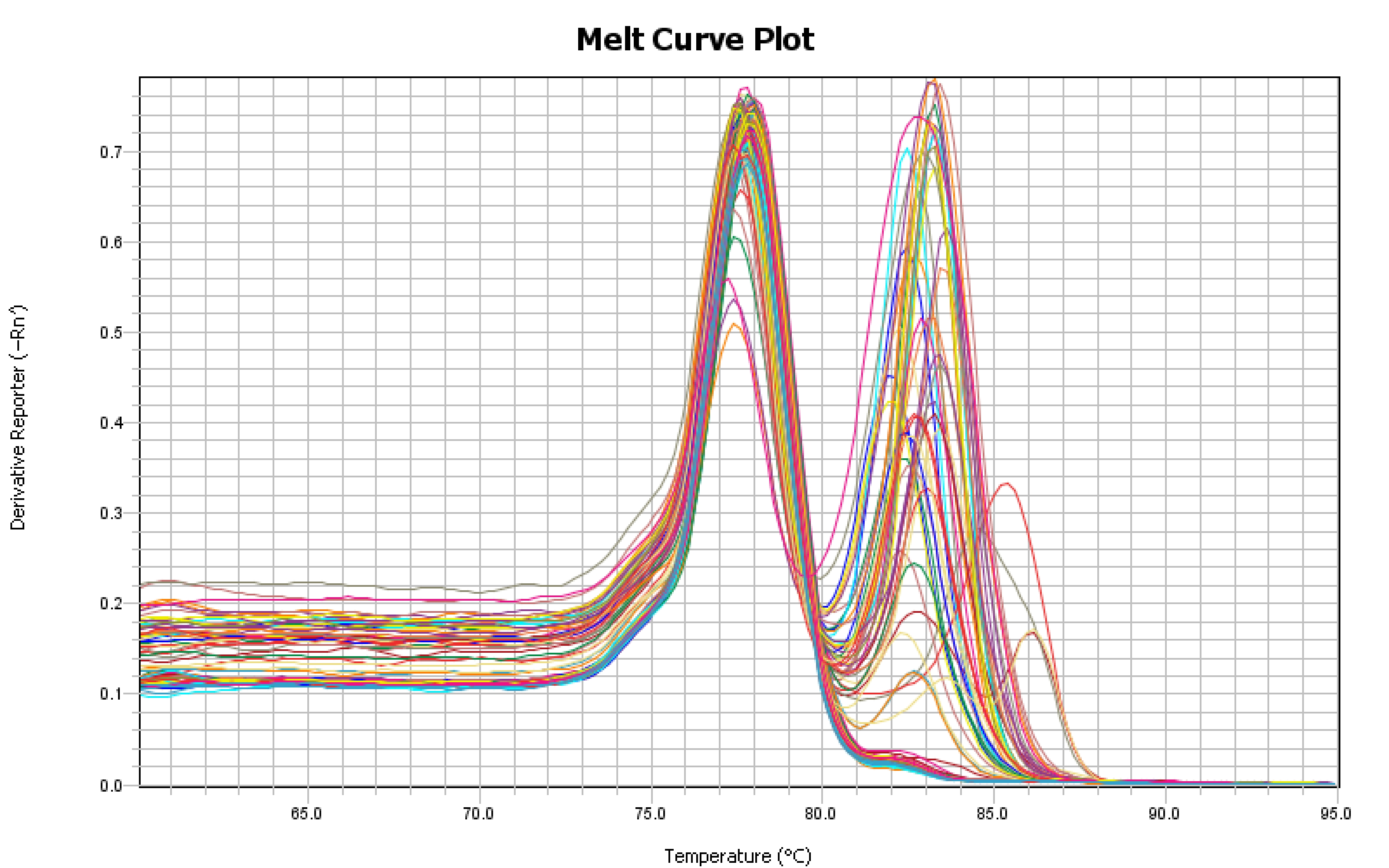


**SnoR14**

**HSP90**


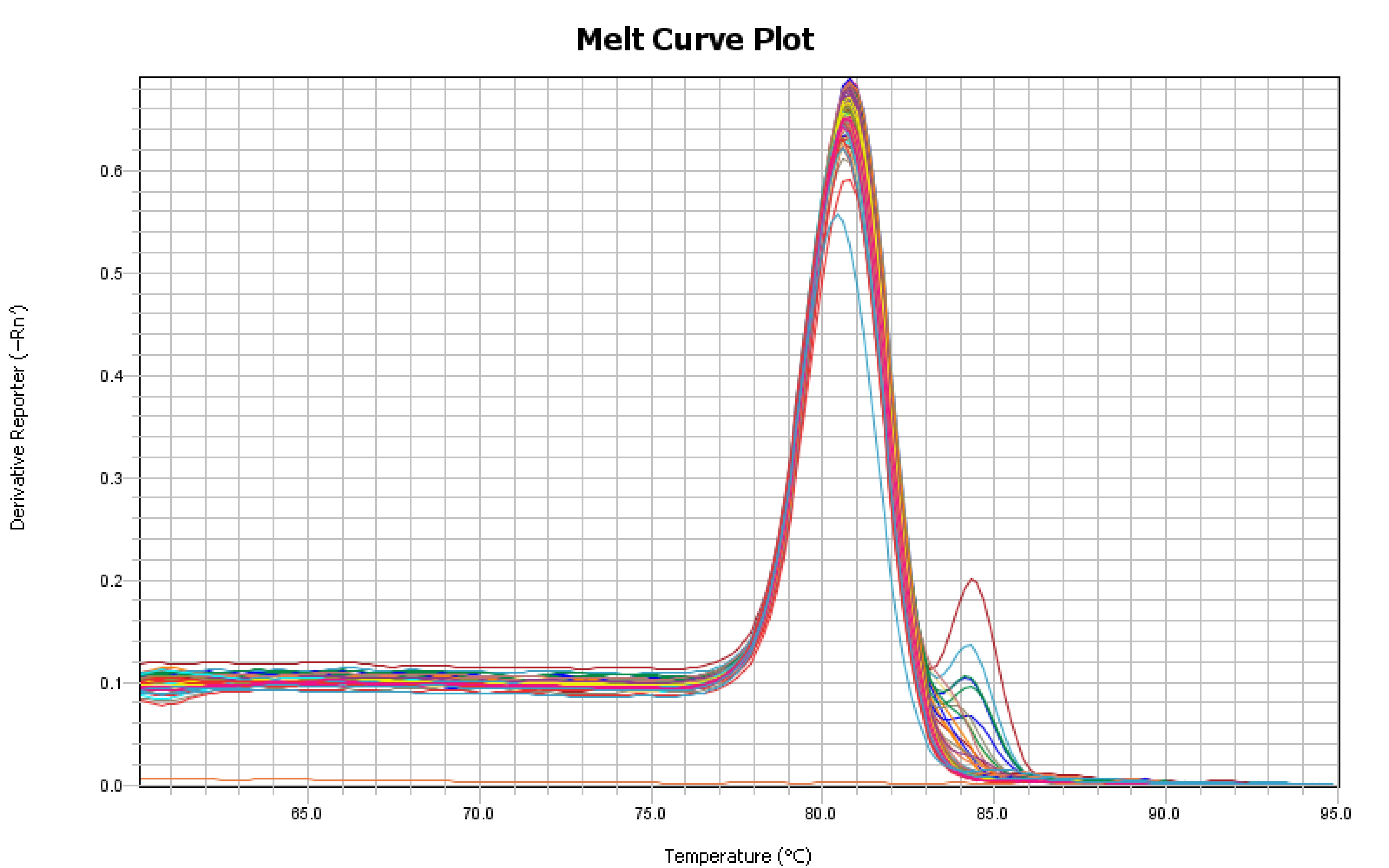

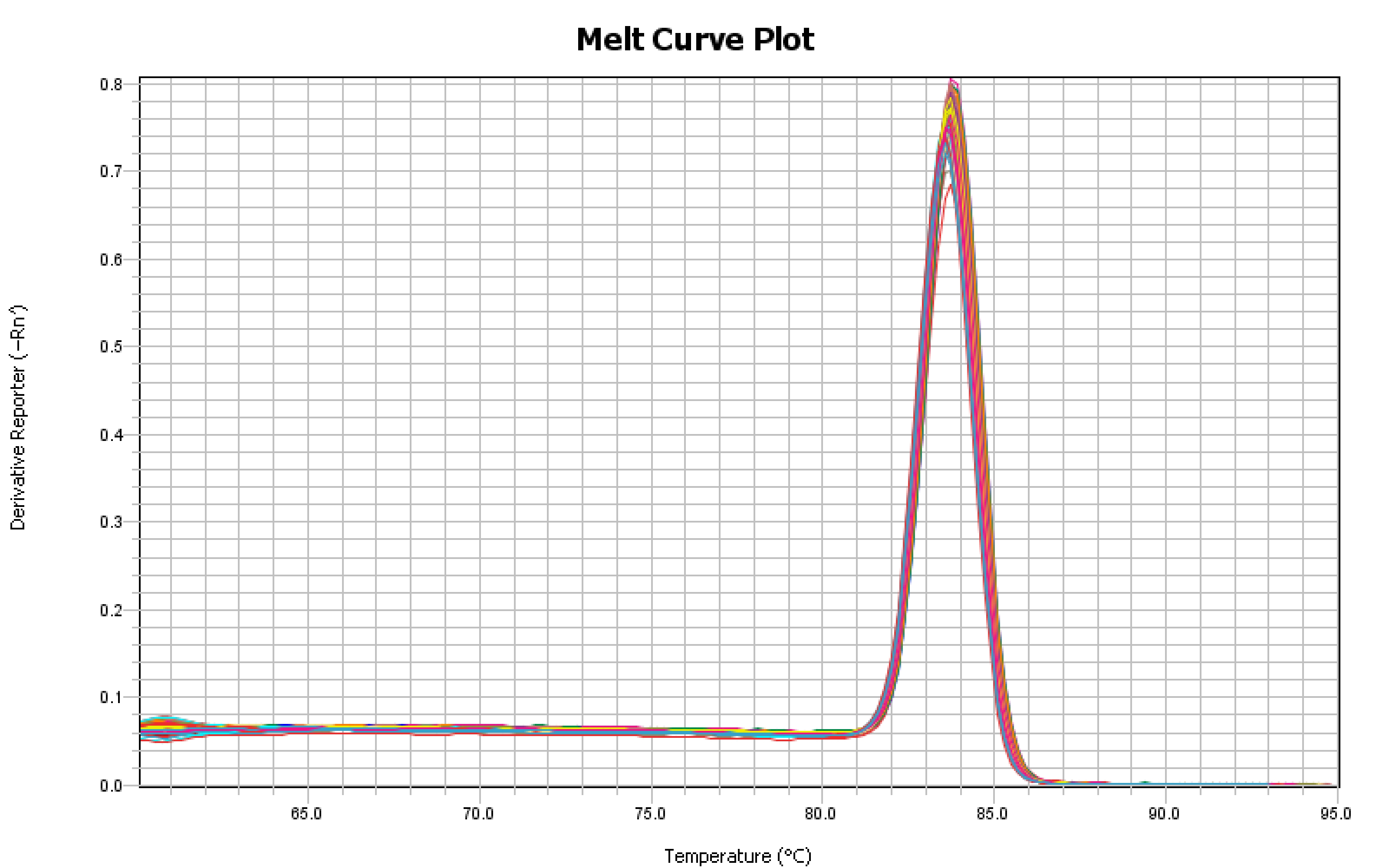


**TCPT**

**SnoR23**


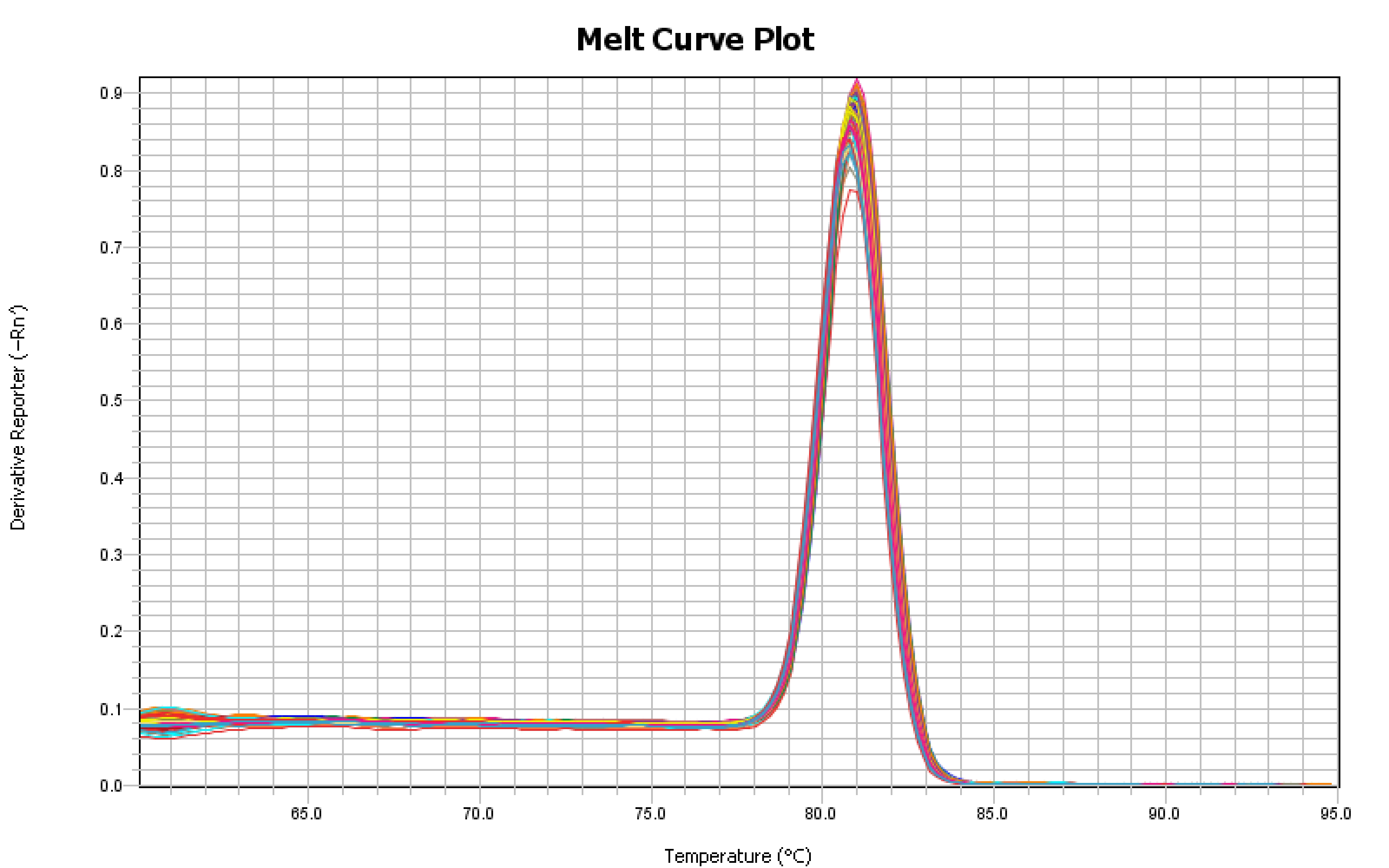


**UBQ**

**S2B Fig. Polyacrylamide gel electrophoresis of PCR product generated from reference gene primers.** Amplicons were run on a 6% TBE polyacrylamide gel.

**
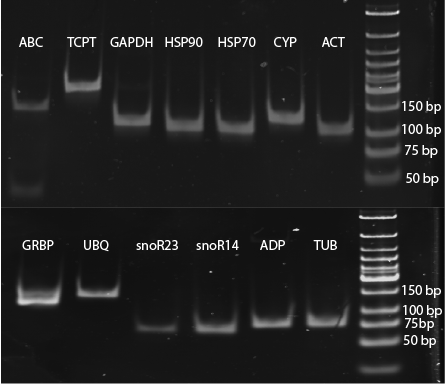
**
