# Supplementary material for: Evaluation and selection of internal reference genes from two- and six-row U.S. malting barley varieties throughout micromalting for use in RT-qPCR
Source: PLoS One. 2018 May 8;13(5):e0196966. doi: 10.1371/journal.pone.0196966 (PMC5940201; doi:10.1371/journal.pone.0196966)
Supplement: S1 Table — (DOCX) [file pone.0196966.s003.docx]

**S1 Table. Malting quality data for two malting varieties used in this study: Conrad (two-row) and Legacy (six-row).**

| **A. Two-Row Barley^a^** | | | | | | | | | | | | | | | |
| --- | --- | --- | --- | --- | --- | --- | --- | --- | --- | --- | --- | --- | --- | --- | --- |
| **Biological Rep** | **Lab Number** | **Crop Year** | **Location** | **Kernel Weight (mg)** | **Barley Color (Agtron)** | **ME (%)** | **Wort Color** | **Barley Protein (%)** | **Wort Protein (%)** | **S/T (%)** | **DP (°ASBC)** | **AA (20°DU)** | **BG (ppm)** | **FAN (ppm)** | **Quality Score** |
| 1 | 2615 | 2015 | Aberdeen, ID | 38.6 | 47 | 79.9 | 3.4 | 13.6 | 5.52 | 44 | 163 | 104.5 | 117 | 291 | 50 |
| 2 | 5582 | 2014 | Morris, MN | 40.7 | 33 | 79.6 | 2.7 | 13.6 | 5.3 | 40 | 167 | 90.9 | 78 | 265 | 51 |
| 3 | 2407 | 2014 | Aberdeen, ID | 37.5 | 36 | 79.3 | 3.9 | 13.8 | 5.46 | 41 | 144 | 93.8 | 74 | 293 | 49 |
| 4 | 5549 | 2014 | Crookston, MN | 38.2 | 43 | 81.2 | 3.9 | 13.1 | 5.73 | 45 | 139 | 92.5 | 183 | 328 | 51 |
| 5 | 5249 | 2015 | Fargo, ND | 37.3 | 46 | 80.9 | 4.2 | 12.9 | 6.12 | 52 | 136 | 95 | 71 | 332 | 50 |
|  |  |  | **Mean** | **38.5** | **41** | **80.2** | **3.6** | **13.4** | **5.63** | **44** | **150** | **95.3** | **105** | **302** | **50** |
|  |  |  | **Min** | **37.3** | **33** | **79.3** | **2.7** | **12.9** | **5.3** | **40** | **136** | **90.9** | **71.4** | **265** | **49** |
|  |  |  | **Max** | **40.7** | **47** | **81.2** | **4.2** | **13.8** | **6.12** | **52** | **167** | **104.5** | **183.5** | **332** | **51** |
|  |  |  |  |  |  |  |  |  |  |  |  |  |  |  |  |
| **B. Six-Row Barley** | | | | | | | | | | | | | | | |
| **Biological Rep** | **Lab Number** | **Crop Year** | **Location** | **Kernel Weight (mg)** | **Barley Color (Agtron)** | **ME (%)** | **Wort Color** | **Barley Protein (%)** | **Wort Protein (%)** | **S/T (%)** | **DP (°ASBC)** | **AA (20°DU)** | **BG (ppm)** | **FAN (ppm)** | **Quality Score** |
| 1 | 5312 | 2013 | Aberdeen, ID | 34.8 | 75 | 81.1 | 2.6 | 13.4 | 6.39 | 50 | 175 | 96.5 | 294 | 324 | 51 |
| 2 | 5313 | 2013 | Morris, MN | 33.1 | 68 | 79.4 | 2.7 | 12 | 5.85 | 53 | 141 | 94.8 | 115 | 308 | 61 |
| 3 | 5314 | 2014 | Aberdeen, ID | 30 | 69 | 79.7 | 2.6 | 13 | 5.95 | 50 | 138 | 92.8 | 192 | 301 | 46 |
| 4 | 5315 | 2012 | Crookston, MN | 33.4 | 47 | 79.3 | 2.6 | 13.6 | 6.35 | 48 | 183 | 98.8 | 162 | 326 | 49 |
| 5 | 5316 | 2012 | Fargo, ND | 30.7 | 61 | 79.4 | 3.1 | 14.9 | 7.09 | 51 | 176 | 104.5 | 133 | 380 | 43 |
|  |  |  | **Mean** | **32.4** | **64** | **79.8** | **2.7** | **13.4** | **6.33** | **50** | **163** | **97.5** | **179** | **328** | **50** |
|  |  |  | **Min** | **30** | **47** | **79.3** | **2.6** | **12** | **5.85** | **48** | **138** | **92.8** | **115.2** | **301** | **43** |
|  |  |  | **Max** | **34.8** | **75** | **81.1** | **3.1** | **14.9** | **7.09** | **53** | **183** | **104.5** | **293.6** | **380** | **61** |

^a^ ME: malt extract; S/T: soluble/total; DP: diastatic power; AA: alpha-amylase; BG: beta-glucan; FAN: free amino nitrogen.
